# Supplementary material for: Neuro-Inflammatory and Behavioral Changes Are Selectively Reversed by Sceletium tortuosum (Zembrin®) and Mesembrine in Male Rats Subjected to Unpredictable Chronic Mild Stress
Source: Cells. 2025 Jul 4;14(13):1029. doi: 10.3390/cells14131029 (PMC12249403; doi:10.3390/cells14131029)
Supplement: Supplementary file 1 [file cells-14-01029-s001.zip › Supplementary material A_Sex effects and female treatment response_Final version_reg.pdf]

SUPPLEMENTARY DATA A: FEMALE WISTAR RATS DATA

Neuroinflammatory and behavioral changes are selectively reversed by *Sceletium tortuosum* (Zembrin®) and mesembrine in male rats subjected to unpredictable chronic mild stress

*Johané Gericke[a], Stephan Steyn[a] Francois Viljoen[a], Brian H. Harvey[a,b,c]**

[a]*Centre of Excellence for Pharmaceutical Sciences, Faculty of Health Sciences, North-West University, Potchefstroom, RSA*

[b]*South African Medical Research Council Unit on Risk and Resilience in Mental Disorders, Department of Psychiatry and Neuroscience Institute, University of Cape Town, Cape Town, RSA*

[c]*The Institute for Mental and Physical Health and Clinical Translation, School of Medicine, Deakin University, Geelong 3220, AUS*

*Corresponding author: Brian.harvey@nwu.ac.za; Tel: +27 18 299 2234

ORCiD of Authors

Johané Gericke: https://orcid.org/0000-0003-0038-3374

Stephan Steyn: https://orcid.org/0000-0002-0023-9711

Francois Viljoen: https://orcid.org/0000-0002-0145-7104

Brian H Harvey: https://orcid.org/0000-0002-9864-7894

1. Introduction

Although UCMS induced bio-behavioural changes in male and female Wistar rats, the overall effect induced in male rats was larger than that observed in female counterparts. Consequently, the intervention effects were also investigated only in male rats (see Article for details). To supplement these findings, the various bio-behavioural parameters of the female Wistar rats are statistically evaluated in this document, as are the intervention effects in the combined group (i.e., irrespective of sex). Moreover, to determine whether any sex-specific differences existed, appropriate statistical analyses are also used to explore this possibility. It is however important to keep in mind that the influence of sex was *not* hypothesized to be a significant contributor and was therefore not factored in during the original power analysis. Consequently, the results reported here must be read with the knowledge that these analyses are underpowered, thereby serving as indicators to be explored in future studies.

2. Statistical methodology

GraphPad Prism was used for all statistical analysis and graphical presentations. All data sets were also screened for outliers using Grubbs' test (α = 0.5). To investigate the bio-behavioural effect of the different interventions on UCMS-exposed female Wistar rats, Kruskal-Wallis and Dunn's multiple comparison tests were used to compare the various parameters of the intervention groups (ESC, ZEM12.5, ZEM25, MES) to the UCMS placebo control group. To determine whether any sex-differences existed, normal two-way ANOVAs[1] were used, with multiple comparison results reported as Bonferroni-adjusted values. For all analyses, statistical significance was set at $p \leq 0.10$ (because of small sample sizes), whilst effect magnitude is expressed as Cohen's *d*-values (with 95% confidence intervals). All in-text values are reported as mean values ± standard deviation. Only statistically significant differences are mentioned in text, with a comprehensive summary of the statistical analyses available in the specified tables.

3. Bio-behavioural effects of treatment - Female rats

3.1. Behavioural parameters (Table S1)

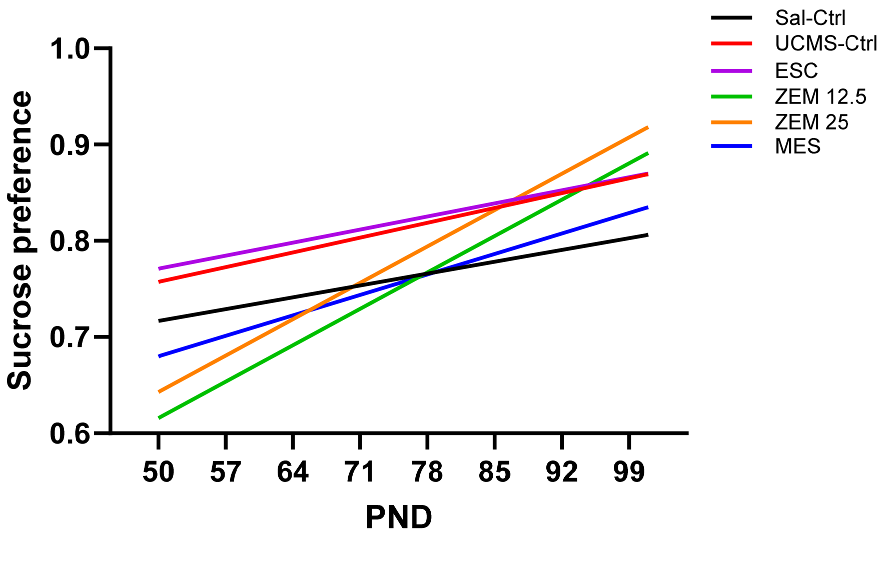

Figure 1. Intervention effects on sucrose preference of UCMS-exposed female rats.
The regression lines indicate the sucrose preference of the different experimental groups over the entire experimental period. All statistical results are summarized in Table S1.

[1] *Sex and UCMS (or treatment) set as influencing factors.*

As summarized in Table S1, baseline (PND50) sucrose preference values in the SPT were comparable between all UCMS-exposed female rat groups ($X^2(4) = 4.08$, $p = 0.39$). Secondly, although the regression line slopes of the different experimental groups were comparable ($F_{5, 228} = 1.71$, $p = 0.13$), only that of Ctrl ($F_{1, 58} = 2.20$, $p = 0.14$) and ESC ($F_{1, 28} = 2.17$, $p = 0.15$) did not deviate statistically from zero, whilst the positive slope of the ZEM12.5 ($R^2 = 0.35$) and ZEM25 ($R^2 = 0.34$) groups were the most obvious. Comparing the mean sucrose preference over time, there was no statistical difference between the Sal+Ctrl (0.76 ± 0.13) and SAL+UCMS (0.82 ± 0.07; $U = 56$, $p = 0.37$, $d = 0.8$ [-0.4; 2.2]) groups, nor between any of the different intervention groups ($X^2(4) = 5.56$, $p = 0.23$).

Interestingly, immobility ($X^2(4) = 12.25$, $p = 0.02$) and swimming ($X^2(4) = 12.33$, $p = 0.02$) time of UCMS-exposed female rats in the FST were altered by the experimental interventions, despite not influencing distance moved in either the OFT ($X^2(4) = 7.99$, $p = 0.09$) or EPM ($X^2(4) = 8.30$, $p = 0.08$). Relative to placebo control UCMS rats, only ZEM12.5 decreased time spent swimming ($p = 0.04$, $d = 1.5$ [0.4; 2.7]) and increased immobility time ($p = 0.02$, $d = 1.6$ [0.5; 2.8]), whilst ESC-administered rats spent less time struggling ($p = 0.05$, $d = 1.34$ [0.3; 2.5]).

None of the parameters, related to anxiety-like behavior of female rats, were influenced by the different experimental interventions in either the EPM or OFT (all p-values > 0.05; Table S1). However, in the BM, the intervention strategies did influence the number of edge explorations (i.e., head dips) ($X^2(4) = 9.23$, $p = 0.06$), with MES increasing edge exploration ($p = 0.04$, $d = 1.43$ [0.4; 2.6]).

3.2. Biological markers (Table S2)

Experimental treatment interventions induced statistically significant changes in cortical PDE4B ($X^2(4) = 14.54$, $p = 0.006$), 5-HIAA ($X^2(4) = 10.11$, $p = 0.04$), DOPAC ($X^2(4) = 13.21$, $p = 0.01$), DOPAC/DA ($X^2(4) = 19.95$, $p = 0.0005$), NA ($X^2(4) = 8.83$, $p = 0.07$), 3-CLT ($X^2(4) = 7.96$, $p = 0.09$), 3-CLT ($X^2(4) = 7.96$, $p = 0.09$), 3-CLT/TYR ($X^2(4) = 13.64$, $p = 0.009$), and GSH ($X^2(4) = 13.60$, $p = 0.009$). In the HC of UCMS-exposed female rats, statistically significant changes were observed in PDE4B ($X^2(4) = 12.02$, $p = 0.17$), 5-HIAA ($X^2(4) = 10.35$, $p = 0.04$), DOPAC ($X^2(4) = 9.38$, $p = 0.05$), NA ($X^2(4) = 9.66$, $p = 0.05$), 3-CLT/TYR ($X^2(4) = 13.03$, $p = 0.01$), GSH ($X^2(4) = 12.56$, $p = 0.01$), GSSG ($X^2(4) = 14.01$, $p = 0.007$), and GSSG/GSH ($X^2(4) = 13.99$, $p = 0.007$).

Relative to UCMS placebo controls, **ESC** increased cortical DOPAC ($p = 0.008$, $d = 1.3$ [0.3; 2.5]) and DOPAC/DA ($p = 0.04$, $d = 1.38$ [0.3; 2.5]) values, and decreased hippocampal GSSG/GSH ($p = 0.002$, $d = 1.16$ [-2.3; -0.1]). **ZEM12.5** increased cortical 5HIAA ($p = 0.01$, $d = 1.39$ [0.3; 2.5]), decreased cortical ($p = 0.04$, $d = 2.19$ [3.7; 0.9] and hippocampal ($p = 0.02$, $d = 1.46$ [2.8; 0.3]) PDE4B, and decreased hippocampal GSH ($p =$

0.01, *d* = 1.5 [2.6; 0.4]), and GSSG (*p* = 0.02, *d* = 1.22 [2.4; 0.2]). **ZEM25** increased cortical 3-CLT/TYR (*p* = 0.06, *d* = 1.1 [0.1; 2.2]) and GSH (*p* = 0.005, *d* = 1.82 [0.7; 3.1]), decreased hippocampal DOPAC (*p* = 0.05, *d* = 0.96 [-2.0; 0.1]), and increased hippocampal 3-CLT/TYR (*p* = 0.03, *d* = 1.73 [0.6; 3.1]). **MES** increased cortical DA turnover (*p* = 0.03, *d* = 1.5 [0.4; 2.7]) and decreased hippocampal PDE4B (*p* = 0.08, *d* = 1.35 [2.6; 0.2]).

3.3. The influence of different treatment strategies on the bio-behavioral parameters of UCMS-exposed female Wistar rats

Firstly, there was an overall improvement in anhedonia-like behavior of UCMS-exposed Wistar rats over time, irrespective of treatment intervention (comparable positive regression line slopes). That said, it is worth noting that this increase in sucrose preference was augmented, specifically by ZEM12.5 and ZEM25, potentially pointing towards some mood enhancing effects in female rats. This apparent pro-hedonic effect of ZEM12.5 over time could be based on its attenuative effects on cortico-hippocampal PDE4B expression (see Article) also observed in the female rats (Table S2), since PDE4B expression was negatively correlated with anhedonia-like behavior (see Article). However, ovarian hormones may also influence taste, which begs the question whether the SPT is indeed a valid and suitable test for female rats [1].

Still, ZEM12.5 induced similar FST-related behavioral alterations in female rats, as reported in male counterparts. To this end, ZEM12.5 increased depressive-like behavior (time spent immobile) and decreased escape-directed behavior (time spent struggling), which may controversially suggest a depressogenic effect [2] – behavior also displayed by ESC-administered female rats, yet not by the ZEM25-administered animals. This apparent depressogenic effect is further highlighted by decreased hippocampal GSH and GSSG (without altering GSSG/GSH), which could indicate an overall suppression of the glutathione pathway, resulting in pro-oxidant effects [3]. That GSH-administration has previously been reported to decrease depressive-like behavior, further supports this possibility [4]. Also, increased cortical 5HIAA can be indicative of depression and anxiety [5], which is relevant here, as ZEM12.5 also increased cortical 5-HIAA concentrations in female Wistar rats. Considered together, despite the apparent pro-hedonic effects of ZEM12.5 observed in female Wistar rats, it may be that such a low dose could potentially induce unwanted neurochemical alterations that would worsen depressive-like behavior. These potentially unwanted effects of ZEM12.5 observed in female rats (as opposed to the potential beneficial effects observed in male rats), could be attributed to female hormones and/or pharmacokinetic and pharmacodynamic differences [6, 7], that could have influenced the biological activity and pharmacological interactions of specific alkaloid constituents in the extract [8].

ZEM25 failed to induce any statistically significant effects in any of the behavioral parameters. On a neurochemical level (summarized in Table S2), ZEM25 increased cortical GSH indicating some antioxidant activity, although it also increased cortico-hippocampal 3-CLT/TYR, which in turn could be indicative of pro-inflammatory effects induced in female rats. Yet, effect size comparisons indicate that ZEM25 had a larger effect on cortical GSH (d = 1.8 [0.7; 3.1]) than 3-CLT/TYR (d = 1.1 [0.1; 2.2]), suggesting that this dose induced a more beneficial antioxidant effect, relative to the suspected pro-inflammatory effect. Its ability to decrease hippocampal DOPAC could possibly contribute to the apparent pro-hedonic effects of this dose over time, although this is not reflected in the individual SPT timepoints or any other markers.

MES only increased edge exploration of the female rats in the BM, indicating possible anxiolytic effects. It also increased cortical DA turnover, which could decrease DA activity and subsequently contribute to the apparent anxiogenic effects [9]. Additionally, MES attenuated hippocampal PDE4B expression but seems to be less potent than ZEM12.5. Since the MES dose used in this study is equivalent to the MES ratio in ZEM12.5 [8] (also see the Article) and it can be concluded that MES contributes to the PDE4 inhibition of the extract, but that synergistic effects from other constituents may increase the potency of the extract – as was also concluded in the male rats [8] (also see the Article). Similarly, it is unlikely that the other observed effects of ZEM12.5 discussed above can be attributed to MES.

Interestingly, ESC only increased sucrose preference on PND92 and decreased struggling in the FST, indicating possible antidepressant- and anxiolytic-like behavioral effects in the female rats. That said, ESC increased cortical DOPAC and DOPAC/DA, possibly indicating decreased DA metabolism and signalling that may point towards a depressogenic effect. On the other hand, ESC appears to induce antioxidant effects based on its ability to decrease hippocampal GSSG/GSH. Previous work supports these results as ESC also induced similar results in the hippocampus of rats exposed to UCMS [10].

4. Bio-behavioural effects of treatment - irrespective of sex

4.1. Behavioral parameters (Table S3)

Next, the bio-behavioral effects of the different intervention strategies are explored, irrespective of sex. As the working hypothesis of this project was made, independent of the influence of sex, it is therefore vital that the sex-specific findings reported here (female rats) and in the Article (male rats) be interpreted against the background of these results.

In terms of sucrose preference, a simple linear regression analysis (Figure), suggested that there were no statistically significant differences between the slopes of the different intervention groups ($F_{5,462} = 2.05$, $p = 0.07$), and that only the slopes of ESC ($F_{1,53} = 4.19$, $p = 0.05$, $R^2 = 0.07$), ZEM12.5 ($F_{1,58} = 26.67$, $p < 0.0001$, $R^2 = 0.32$), and ZEM25 ($F_{1,58} = 9.18$, $p = 0.004$, $R^2 = 0.14$) administered groups deviated statistically from zero. Importantly, although a repeated measures ANOVA indicated that there was no statistically significant interaction between time and intervention ($F_{16,263} = 1.21$, $p = 0.26$), the main effect of time did have a significant influence on sucrose preference ($F_{3.172,208.6} = 10.54$, $p < 0.0001$), thereby supporting the positive pooled regression line slope of the different intervention groups. It is also important to note that there were no baseline sucrose preference differences between these experimental groups ($X^2(4) = 3.76$, $p = 0.44$).

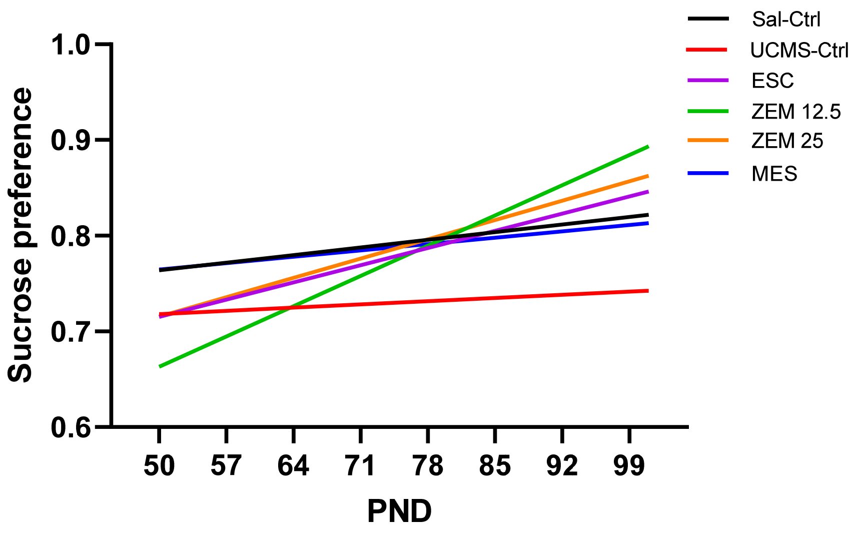

Figure S2. Effect of treatment in on sucrose preference (SPT) over time in both sexes combined

Irrespective of sex, treatment interventions (Table S3) altered time spent immobile ($X^2(4) = 20.91$, $p = 0.0003$) and swimming ($X^2(4) = 21.72$, $p = 0.0002$) in the FST, without influencing distance moved in the OFT ($X^2(4) = 8.69$, $p = 0.07$). Time spent in the center zone of the OFT was, however, affected by treatment ($X^2(4) = 8.69$, $p = 0.002$). Compared to the UCMS controls, **ZEM12.5** decreased swimming ($p = 0.002$, $d = 1.24$ [2.0; 0.5]) and increased immobility ($p = 0.002$, $d = 1.33$ [0.6; 2.1]) time in the FST, whilst **ESC** decreased center time duration ($p = 0.009$, $d = 1.35$ [2.2; 0.6]) in the OFT.

4.2. Biological markers (Table S4)

Irrespective of sex, treatment interventions induced statistically significant effects in cortical PDE4B ($X^2(4) = 25.14$, $p = <0.0001$), 5-HIAA ($X^2(4) = 23.19$, $p = 0.0001$), 5-HIAA/5-HT ($X^2(4) = 10.65$, $p = 0.03$, DA ($X^2(4) =$

16.84, $p = 0.002$), DOPAC/DA ($X^2(4) = 23.01$, $p = 0.0001$), 3-CLT/TYR ($X^2(4) = 15.73$, $p = 0.003$), GSSG ($X^2(4) = 10.78$, $p = 0.03$), GSSG/GSH ($X^2(4) = 12.26$, $p = 0.02$). In the hippocampus, PDE4B ($X^2(4) = 27.47$, $p = <0.0001$), 5-HIAA ($X^2(4) = 10.24$, $p = 0.04$), NA ($X^2(4) = 16.39$, $p = 0.003$), GSSG ($X^2(4) = 21.03$, $p = 0.0003$), and GSSG/GSH ($X^2(4) = 16.89$, $p = 0.002$) were altered by the pharmacological interventions, as were plasma TNF-α levels ($X^2(4) = 14.76$, $p = 0.01$).

Relative to the UCMS control rats, **ESC**, independent of sex, increased cortical DOPAC/DA ($p = 0.03$, $d = 0.8$ [0.1; 1.6]), and decreased hippocampal GSSG ($p = 0.02$, $d = 0.9$ [1.7; 0.2]) and GSSG/GSH ($p = 0.01$, $d = 1.0$ [1.8; 0.3]). **ZEM12.5** decreased cortical PDE4B ($p < 0.0001$, $d = 2.2$ [1.3; 3.3]), and increased 5-HIAA ($p = 0.01$, $d = 1.2$ [0.5; 2.0]) and 5-HIAA/5-HT ($p = 0.04$, $d = 1.2$ [0.5; 2.0]). It also decreased hippocampal PDE4B ($p < 0.0001$, $d = 2.0$ [1.1; 3.0]) and GSSG ($p = 0.04$, $d = 0.8$ [0.1; 1.5]), and increased 5-HIAA ($p = 0.03$, $d = 1.1$ [0.4; 1.9]). In the frontal cortex, **MES** decreased DA ($p = 0.03$, $d = 0.8$ [0.1; 1.5]), and increased DOPAC/DA ($p = 0.01$, $d = 1.1$ [0.4; 1.8]), and 3-CLT/TYR ($p = 0.01$, $d = 1.1$ [0.4; 1.8]) values. MES further decreased hippocampal PDE4B ($p = 0.001$, $d = 1.6$ [0.8; 2.5]) and NA ($p = 0.01$, $d = 1.0$ [0.3; 1.8]).

4.3. Effects of UCMS and treatment on both sexes combined

In the Article, we observed minimal effects of the UCMS on the combined male and female group, save for increased swimming and decreased immobility in the FST, along with increased locomotor activity in the OFT. At first glance it seemed as if these results indicated that the UCMS failed to induce depression- and/or anxiety-like bio-behavioral changes. However, when distinguishing between male and female rats, behavioral alterations were noted (see above and in the Article). For instance, the mentioned increase in locomotor activity (as observed in the OFT) was only true for UCMS-exposed male Wistar rats, when analyzed separately. Interestingly, except for an increase in cortical 3-CLT/TYR (indicative of pro-inflammatory responses and oxidative stress [11]), no other statistically significant differences were observed in any of the biological markers when ignoring the influence of sex.

Regarding the effects of treatment in the combined sex group (Table S3 and Table S4), **ESC** appeared to increase anxiety-like behavior in the OFT by increasing time spent in the corners and decreasing time spent in the center zone of the maze The former was also observed in ESC-administered male rats, suggesting that the ESC could induce anxiety-like behavior, possibly due to a higher dose (20 mg/kg). That ESC also increased cortical DA turnover, could partly have contributed to the lack of differences in sucrose preference, as increased 5-HT concentrations could decrease DA activity via complex mechanisms and

pathways [12]. ESC-administration did, however, induce some antioxidant and anti-inflammatory activity by decreasing hippocampal GSSG and plasma TNF-α, which coincides with literature.

ZEM12.5 reversed the UCMS-induced increased mobility in the FST and OFT, and increased sucrose preference on PND101, thus indicating anxiolytic-like and some antidepressant-like effects. This also aligns with the male and female rat responses. In terms of neurochemistry, ZEM12.5 decreased cortical and hippocampal PDE4B, emphasizing the potent inhibitory effects of this dose, despite UCMS failing to increase PDE4B in the frontal cortex. The combined sex group also presented with increased cortical and hippocampal 5-HIAA and cortical serotonin turnover (5-HIAA/5-HT), which could be skewed due to the female response to ZEM12.5, described above. Lastly, ZEM12.5 decreased hippocampal GSSG indicating antioxidant effects, echoing the described female response.

ZEM25 did not cause ant bio-behavioral changes, when the influence of sex was ignored, despite altering analyzed parameters in the isolated male and female cohorts.

Finally, **MES** only increased sucrose preference on PND78 in the combined group, as reported for the male rat cohort. No other behavioral parameters were affected. Interestingly, MES only managed to decrease hippocampal PDE4B in the combined group, further proving that MES only contributes to the potent PDE4 inhibition of ZEM12.5 and is not the only role player in this important mechanism of action of the *Sceletium tortuosum* extract. Counterintuitively, the combined sex group displayed simultaneous increased DA and DA turnover after MES exposure. However, these results are skewed by the increased DA turnover of female rats, and the increase in cortical DA seen in the male rats – altogether pointing towards transient pro-hedonic effects in the male rats. Additionally, MES increased cortical tyrosine turnover (3-CLT/TYR) in the combined group, indicating inflammation and/or oxidative stress, while unexpectedly also decreasing hippocampal NA, despite not being statistically significant in either sex cohort. Yet, this decrease in hyperarousal was only seen in ZEM12.5-administered animals, and not in those exposed to MES. Reminiscent of the less pronounced PDE4 inhibition of MES, compared to ZEM12.5, the serotonergic effect of MES could be masked or inhibited by other alkaloids in the extract, while the noradrenergic effect remains with MES alone in the combined sex group. Alternatively, MES may decrease NA via an unknown mechanism since none of its known mechanisms are directly related to NA inhibition. This suspicion further emphasizes the value of investigating individual and combined effects of *Sceletium tortuosum* constituents to gain a better understanding of the mechanisms of this plant and to prevent possible side effects or interactions.

5. Statistical investigation into potential sex differences

5.1. UCMS-induced bio-behavioral effects (male *vs.* female) (Table S5 and S6)

To statistically explore the influence of sex on the different behavioral parameters, normal two-way ANOVAs were used, with sex and UCMS and intervention set as influencing factors. These results are summarized in Table 5 and S**Parameter**	**2-Way ANOVA: *p*-values**			**Group differences**	
	**Interaction**	**Sex**	**Stress**		
**SUCROSE PREFERENCE TEST (SPT)**					
**Sucrose preference PND 50**	0.2098	0.8261	0.2439	ns	
**Sucrose preference PND 64**	**0.0086****	0.1774	0.3214	Males:UCMS vs. Females:UCMS	*p* = 0.0335*
**Sucrose preference PND 78**	**0.0078****	0.6005	0.1923	Males:Ctrl vs. Males:UCMS	*p* = 0.0341*
**Sucrose preference PND 92**	**0.0089****	0.3222	0.4616	ns	
**Sucrose preference PND 101**	**0.0248***	**0.0065****	**0.0309***	Males:Ctrl vs. Males:UCMS<br>Males:UCMS vs. Females:Ctrl<br>Males:UCMS vs. Females:UCMS	*p* = 0.0145*<br>*p* = 0049**<br>*p* = 0.004**
**OPEN FIELD TEST (OFT)**					
**Distance (cm)**	0.1715	0.7784	**0.0004*****	Males:Ctrl vs. Males:UCMS<br>Males:UCMS vs. Females:Ctrl	*p* = 0.0039**<br>*p* = 0.0356*
**Center duration (s)**	0.6360	0.7740	0.2396	ns	

**Corner duration (s)**	0.2538	**0.0014****	0.6275	Males:UCMS vs. Females:UCMS	0.0048*
**FORCED SWIM TEST (FST)**					
**Swimming (s)**	0.2643	0.5777	**0.0004*****	Males:Ctrl vs. Males:UCMS	0.0022**
**Immobility (s)**	0.6810	0.4809	**0.0002*****	Males:Ctrl vs. Males:UCMS<br>Females: Ctrl vs Females: UCMS	0.0056**<br>0.0264*
**Struggling (s)**	**0.0049****	0.5060	0.3135	Females:Ctrl vs. Females:UCMS	0.0432*
**BARNES MAZE (BM)**					
**Distance (cm)**	0.6816	0.3406	0.7384	ns	
**Primary error rate**	**0.0192***	0.6148	0.0729	Males:Ctrl vs. Males:UCMS	
**Edge exploration (head dips)**	**0.0072****	0.5865	0.1246	Females:Ctrl vs. Females:UCMS	
**Outer/inner duration**	0.5619	0.0511	**0.0400***	ns	

Table S6, with an overview of the most significant findings, discussed below.

Of the fifteen behavioral parameters (Table S5) analyzed, seven (sucrose preference on PND64 and PND101, distance moved and corner duration in the OFT, struggling in the FST, and primary error rate and edge exploration in the BM) revealed statistically significant interactions between sex and UCMS, suggesting	**2-Way ANOVA: *p*-values**			**Group differences**
	**Interaction**	**Sex**	**Stress**	

that less than half (i.e., 47 %) of the analyzed parameters could potentially highlight sex differences. Furthermore, of the thirty neurochemical markers analyzed (**Parameter**					
**SUCROSE PREFERENCE TEST (SPT)**					
**Sucrose preference PND 50**	0.2098	0.8261	0.2439	ns	
**Sucrose preference PND 64**	**0.0086****	0.1774	0.3214	Males:UCMS vs. Females:UCMS	*p* = 0.0335*
**Sucrose preference PND 78**	**0.0078****	0.6005	0.1923	Males:Ctrl vs. Males:UCMS	*p* = 0.0341*
**Sucrose preference PND 92**	**0.0089****	0.3222	0.4616	ns	
**Sucrose preference PND 101**	**0.0248***	**0.0065****	**0.0309***	Males:Ctrl vs. Males:UCMS<br>Males:UCMS vs. Females:Ctrl<br>Males:UCMS vs. Females:UCMS	*p* = 0.0145*<br>*p* = 0049**<br>*p* = 0.004**
**OPEN FIELD TEST (OFT)**					
**Distance (cm)**	0.1715	0.7784	**0.0004*****	Males:Ctrl vs. Males:UCMS<br>Males:UCMS vs. Females:Ctrl	*p* = 0.0039**<br>*p* = 0.0356*
**Center duration (s)**	0.6360	0.7740	0.2396	ns	
**Corner duration (s)**	0.2538	**0.0014****	0.6275	Males:UCMS vs. Females:UCMS	0.0048*
**FORCED SWIM TEST (FST)**					
**Swimming (s)**	0.2643	0.5777	**0.0004*****	Males:Ctrl vs. Males:UCMS	0.0022**
**Immobility (s)**	0.6810	0.4809	**0.0002*****	Males:Ctrl vs. Males:UCMS<br>Females: Ctrl vs Females: UCMS	0.0056**<br>0.0264*
**Struggling (s)**	**0.0049****	0.5060	0.3135	Females:Ctrl vs. Females:UCMS	0.0432*
**BARNES MAZE (BM)**					
**Distance (cm)**	0.6816	0.3406	0.7384	ns	
**Primary error rate**	**0.0192***	0.6148	0.0729	Males:Ctrl vs. Males:UCMS	
**Edge exploration (head dips)**	**0.0072****	0.5865	0.1246	Females:Ctrl vs. Females:UCMS	
**Outer/inner duration**	0.5619	0.0511	**0.0400***	ns	

Table S6), only 10 % (cortical 5-HT, 5-HIAA/5-HT, and DOPAC) were influenced by the interaction between sex and UCMS. Overall, 22 % of all analyzed parameters could point towards stress-induced sex differences. This objectively small prevalence of statistically significant interactions therefore supports the working hypothesis that UCMS is an effective model to induce bio-behavioral alterations, independent of sex. Read together with the findings reported in the Article, the hypothesis that although male and female rats are affected by UCMS, the effect may be larger in male rats, remains a valid topic to explore in future studies.

5.2. Intervention-induced neurochemical effects (male *vs*. female) (Tables S7 and S8)

From the fifteen behavioral parameters analyzed (Table S7), only two (13 %; PND78 and 101 of the SPT) statistically significant interactions were identified, indicating potential different sex responses to the investigated pharmacological interventions. On the other hand, seven statistically significant interactions were identified from the thirty neurochemical markers analyzed (

Table S8), indicating potential sex differences in 23 % of neurochemical markers (FC: 5-HIAA, NA, 3-CLT/TYR, GSH; HC: 5-HIAA/5-HT, GSSG/GSH; Plasma IL-10). Overall, only 20 % of treatment responses could potentially highlight sex-specific sensitivity. In terms of specific treatment prevalence (ZEM25 (38 %), ZEM12.5 (23 %), ESC (23 %), and MES (15 %)), ZEM25 induced the most sex*treatment interactions, thereby underlining the importance of future confirmatory studies, as well as investigation into the possible mechanisms of these supposed sex-specific reactions. Either way, based on the low prevalence of said two-way interactions (i.e., 20 %), our approach and conclusion in the Article is justified and supported, in that sex-specific effects does not appear to be statistically relevant, yet is a factor that could be further investigated in prospective studies. In fact, that we were able to identify some sex differences in terms of treatment response, emphasizes the value of including females in preclinical studies to better equip researchers to develop individualized medication, and improve translatability.

6. Implications of sex differences in research

One of the problems in current research is the underrepresentation of the female population in preclinical studies [13]. Most of the time, female animals are omitted due to the variable effects of fluctuating female hormones on experimental outcomes [14], and consequent cost implications [15]. Still, using only male animals could give an inaccurate clinical representation, and consequently reduce translatability [13, 15]. Ultimately, this could impair the development of personalized pharmaceutical treatment for women [15].

Literature often suggests that females are generally more stress-sensitive and are almost twice as likely to develop depression, relative to male counterparts [7, 16-18]. However, the current and other UCMS studies have indicated the opposite [19, 20], with male Wistar rats actually being more sensitive to the detrimental effects of UCMS and presenting with an anxious-depressive-like phenotype, whilst female Wistar rats appeared to be less affected – at least in terms of an MDD-relatable bio-behavioral profile.

Despite these supposed sex differences, a meta-analysis by Becker and colleagues [15] reported no significant increase in variability with the inclusion of female subjects, thereby advocating for the inclusion of both male and female subjects. However, as alluded to earlier, such an inclusion could potentially impact, amongst others, the financial aspects of a project. It is therefore noteworthy that Beery [14] suggested that adjustment of statistical analyses by adjusting factorial designs and comparing effect sizes to evaluate sex differences, can be done without increasing sample sizes. The author further claimed that if evaluating sex differences is a primary goal, increased sample sizes would improve detection of interactions. Yet, if it is a secondary goal, the statistical analyses mentioned would suffice with no need to increase sample sizes. Considered together, although the current study did identify certain sex differences, it must be re-emphasized that said investigation did not form part of the original hypothesis and study design. Consequently, the statistical power for such investigations was insufficient (i.e., 25 % generated power), yet a valuable departing point for follow-up studies.

7. Conclusion

UCMS and treatment induced some statistically significant sex differences, however the prevalence of these effects was very low, suggesting that sex did not have a statistically significant effect on either UCMS or treatment responses. Detailed analyses did however suggest that although UCMS induced sex-independent bio-behavioral alterations, the effect was larger in male Wistar rats. In this regard, male rats presented with a bio-behavioral profile reminiscent of anxious depression, whilst the female counterparts appeared to present with more anxiety-like characteristics, without any depressive-like components (anhedonia). Still, biological differences in the sexes can influence responses to stress and treatment and should be investigated in the future using larger sample sizes. As for pharmacological interventions, the highest prevalence of potential sex-dependent responses was observed in the ZEM25-administered group.

8. References

1. Franceschelli, A., Herchick, S., Thelen, C., Papadopoulou-Daifoti, Z., and Pitychoutis, P.M., Sex differences in the chronic mild stress model of depression. *Behavioural Pharmacology*, 2014. **25**(5 and 6): p. 372-383.
2. Gericke, J., Lekhooa, M., Steyn, S.F., Viljoen, A.M., and Harvey, B.H., An acute dose-ranging evaluation of the antidepressant properties of Sceletium tortuosum (Zembrin®) versus escitalopram in the Flinders Sensitive Line rat. *Journal of Ethnopharmaclology,* 2021, p. 114550.
3. Lushchak, V.I., Glutathione homeostasis and functions: potential targets for medical interventions. *Journal of amino acids*, 2012. **2012**(1): p. 736837.
4. Rosa, J.M., Dafre, A.L., and Rodrigues, A.L.S., Antidepressant-like responses in the forced swimming test elicited by glutathione and redox modulation. *Behavioural Brain Research*, 2013. **253**: p. 165-172.
5. Andrews, P.W., Bharwani, A., Lee, K.R., Fox, M., and Thomson Jr, J.A., Is serotonin an upper or a downer? The evolution of the serotonergic system and its role in depression and the antidepressant response. *Neuroscience & Biobehavioral Reviews*, 2015. **51**: p. 164-188.
6. Schwartz, J.B., The influence of sex on pharmacokinetics. *Clinical pharmacokinetics*, 2003. **42**(2): p. 107-121.
7. Konkle, A.T.M., Baker, S.L., Kentner, A.C., Barbagallo, L.S.-M., Merali, Z., and Bielajew, C., Evaluation of the effects of chronic mild stressors on hedonic and physiological responses: sex and strain compared. *Brain Research*, 2003. **992**(2): p. 227-238.
8. Gericke, J., Harvey, B.H., Pretorius, L., Ollewagen, T., Benecke, R.M., and Smith, C., Sceletium tortuosum-derived mesembrine significantly contributes to the anxiolytic effect of Zembrin®, but its anti-depressant effect may require synergy of multiple plant constituents. *Journal of Ethnopharmacology*, 2024. **319**: p. 117113.
9. Zarrindast, M.-R. and Khakpai, F., The modulatory role of dopamine in anxiety-like behavior. *Archives of Iranian medicine*, 2015. **18**(9): p. 0-0.
10. Dionisie, V., Ciobanu, A.M., Toma, V.A., Manea, M.C., Baldea, I., Olteanu, D., Sevastre-Berghian, A., Clichici, S., Manea, M., and Riga, S., Escitalopram targets oxidative stress, caspase-3, BDNF and MeCP2 in the hippocampus and frontal cortex of a rat model of depression induced by chronic unpredictable mild stress. *International journal of molecular sciences*, 2021. **22**(14): p. 7483.
11. Mohiuddin, I., Chai, H., Lin, P.H., Lumsden, A.B., Yao, Q., and Chen, C., Nitrotyrosine and Chlorotyrosine: Clinical Significance and Biological Functions in the Vascular System. *Journal of Surgical Research*, 2006. **133**(2): p. 143-149.
12. Courtiol, E., Menezes, E.C., and Teixeira, C.M., Serotonergic regulation of the dopaminergic system: Implications for reward-related functions. *Neuroscience & Biobehavioral Reviews*, 2021. **128**: p. 282-293.
13. Allegra, S., Chiara, F., Di Grazia, D., Gaspari, M., and De Francia, S., Evaluation of Sex Differences in Preclinical Pharmacology Research: How Far Is Left to Go? *Pharmaceuticals*, 2023. **16**(6): p. 786.
14. Beery, A.K., Inclusion of females does not increase variability in rodent research studies. *Current Opinion in Behavioral Sciences*, 2018. **23**: p. 143-149.
15. Becker, J.B., Prendergast, B.J., and Liang, J.W., Female rats are not more variable than male rats: a meta-analysis of neuroscience studies. *Biology of sex differences*, 2016, **7**(1): p. 1-7.
16. Rincón-Cortés, M., Herman, J.P., Lupien, S., Maguire, J., and Shansky, R.M., Stress: Influence of sex, reproductive status and gender. *Neurobiology of stress*, 2019. **10**: p. 100155.
17. Ter Horst, J.P., de Kloet, E.R., Schächinger, H., and Oitzl, M., Relevance of stress and female sex hormones for emotion and cognition. *Cellular and molecular neurobiology*, 2012. **32**: p. 725-735.
18. Dalla, C., Antoniou, K., Kokras, N., Drossopoulou, G., Papathanasiou, G., Bekris, S., Daskas, S., and Papadopoulou-Daifoti, Z., Sex differences in the effects of two stress paradigms on dopaminergic neurotransmission. *Physiology & Behavior*, 2008. **93**(3): p. 595-605.

19. Dalla, C., Antoniou, K., Drossopoulou, G., Xagoraris, M., Kokras, N., Sfikakis, A., and Papadopoulou-Daifoti, Z., Chronic mild stress impact: Are females more vulnerable? *Neuroscience*, 2005. **135**(3): p. 703-714.
20. Foglio, B. and Panzica, G., Sexually dimorphic effects of unpredictable chronic mild stress (UCMS) in a murine model of depression and anxiety, in Poster Session 2, 7th September 2013-poster number P255. 2013. p. 1-1.

SUPPLEMENTARY MATERIAL

Table S1. Summary of results for the behavioural tests presented for each treatment (Rx) group for females alone. p ≤ 0.1. Outliers indicated as n^{O} (outlier not removed) or n^{R} (outlier removed). 95% CI – 95% confidence intervals; ESC – Escitalopram; MES – Mesembrine; PND – Postnatal day; SD – standard deviation; UCMS – Saline-treated stress group; ZEM 12.5 and 25 - Zembrin®

Parameter	Kruskal-Wallis test	Rx Group	n	Mean ± SD	*p*-value	*d* [95 % CI]
**SUCROSE PREFERENCE TEST (SPT)**						
**Sucrose preference PND 50**	$X^2(4) = 4.08$, $p = 0.3947$	**UCMS**	12	0.72 ± 0.22		
		**ESC**	6	0.68 ± 0.18	>0.9999	-0.17 [-1.2; 0.8]
		**ZEM 12.5**	6	0.59 ± 0.21	0.4050	-0.57 [-1.6; 0.4]
		**ZEM 25**	6	0.61 ± 0.22	0.5220	-0.46 [-1.5; 0.5]
		**MES**	6	0.59 ± 0.25	0.8098	-0.53 [-1.6; 0.5]
**Sucrose preference PND 64**	**$X^2(4) = 8.35$, $p = 0.0795$**	**UCMS**	12°	0.84 ± 0.10		
		**ESC**	6	0.87 ± 0.06	>0.9999	0.33 [-0.7; 1.3]
		**ZEM 12.5**	6°	0.71 ± 0.19	**0.0759**	-0.89 [-2.0; 0.1]
		**ZEM 25**	6°	0.73 ± 0.23	0.4518	-0.67 [-1.7; 0.3]
		**MES**	6	0.81 ± 0.04	0.5284	-0.25 [-1.2; 0.7]
**Sucrose preference PND 78**	$X^2(4) = 7.33$, $p = 0.1193$	**UCMS**	12°	0.84 ± 0.09		
		**ESC**	6°	0.90 ± 0.16	0.1594	0.5 [-0.5 1.5]
		**ZEM 12.5**	6	0.82 ± 0.06	>0.9999	-0.25 [-1.2; 0.7]
		**ZEM 25**	6	0.85 ± 0.03	>0.9999	0.14 [-0.8; 1.1]
		**MES**	6	0.83 ± 0.06	>0.9999	-0.11 [-1.1; 0.9]
**Sucrose preference PND 92**	**$X^2(4) = 12.29$, $p = 0.0153$***	**UCMS**	12	0.83 ± 0.05		
		**ESC**	6	0.90 ± 0.02	**0.0564**	**1.34 [0.3; 2.5]**
		**ZEM 12.5**	6	0.80 ± 0.13	>0.9999	-0.37 [-1.4; 0.6]
		**ZEM 25**	6	0.87± 0.04	0.8491	0.76 [-0.2; 1.8]
		**MES**	6	0.79 ± 0.07	0.8260	-0.78 [-1.8; 0.2]
**Sucrose preference PND 101**	**$X^2(4) = 14.57$, $p = 0.0057$****	**UCMS**	12	0.86 ± 0.07		
		**ESC**	6	0.78 ± 0.06	0.1065	**-1.17 [-2.3; -0.2]**
		**ZEM 12.5**	6	0.90 ± 0.03	>0.9999	0.54 [-0.4; 1.6]
		**ZEM 25**	6°	0.89 ± 0.08	>0.9999	0.33 [-0.7; 1.3]
		**MES**	6	0.79 ± 0.03	0.1463	-1.04 [-2.1; 0.0]
**FORCED SWIM TEST (FST)**						
**Swimming (s)**	**$X^2(4) = 12.33$, $p = 0.0151$***	**UCMS**	12	88.26 ± 37.02		
		**ESC**	6	80.70 ± 46.16	>0.9999	-0.18 [-1.2; 0.8]
		**ZEM 12.5**	6	39.04 ± 13.92	**0.0362**	**-1.48 [-2.7; -0.4]**
		**ZEM 25**	6	101.7 ± 30.53	>0.9999	0..37 [-0.6; 1.4]
		**MES**	6	104.5 ± 26.22	>0.9999	0.46 [-0.5; 1.5]
**Immobility (s)**	**$X^2(4) = 12.25$, $p = 0.0156$***	**UCMS**	12	194.6 ± 40.64		
		**ESC**	6	216.2 ± 45.09	>0.9999	0.49 [-0.5; 1.5]
		**ZEM 12.5**	6	253.5 ± 13.17	**0.0160**	**1.62 [0.5; 2.8]**
		**ZEM 25**	6	185.7 ± 25.12	>0.9999	-0.23 [-1.2; 0.7]

		**MES**	6	180.4 ± 24.94	>0.9999	-0.37 [-1.4; 0.6]
**Struggling (s)**	**X²(4) = 8.38, *p* = 0.0785***	**UCMS**	12	17.34 ± 14.37		
		**ESC**	6	2.856 ± 2.529	**0.0506**	**-1.15 [-2.3; -0.1]**
		**ZEM 12.5**	6	7.609 ± 9.123	0.5224	-0.72 [-1.8; 0.3]
		**ZEM 25**	6	12.04 ± 10.52	>0.9999	-0.38 [-1.4; 0.6]
		**MES**	6	15.44 ± 2.898	>0.9999	-0.15 [-1.1; 0.8]
**OPEN FIELD TEST (OFT)**						
**Distance moved (cm)**	**X²(4) = 7.99, *p* = 0.0916***	**UCMS**	12	9132 ± 1261		
		**ESC**	6	8844 ± 693	>0.9999	-0.57 [-0.3; 0.2]
		**ZEM 12.5**	6	7692 ± 1358	0.1529	**-1.06 [-2.2; -0.1]**
		**ZEM 25**	6	9657 ± 1229	>0.9999	-0.08 [-0.8; 0.6]
		**MES**	6	9494 ± 1239	>0.9999	-0.36 [-1.1; 0.3]
**Center duration (s)**	**X²(4) = 9.74, *p* = 0.0450***	**UCMS**	12	60.23 ± 17.07		
		**ESC**	6	42.35 ± 10.37	0.1308	**-1.11 [-2.2; -0.1]**
		**ZEM 12.5**	6	47.11 ± 28.99	0.7150	-0.58 [-1.6; 0.4]
		**ZEM 25**	6	59.35 ± 16.18	>0.9999	-0.05 [-1.0; 0.9]
		**MES**	6	66.29 ± 6.653	>0.9999	0.39 [-0.6; 1.4]
**Corner duration (s)**	X²(4) = 6.99, *p* = 0.1361	**UCMS**	12	84.93 ± 14.51		
		**ESC**	6	100.2 ± 17.78	0.2264	0.93 [-0.1; 2.0]
		**ZEM 12.5**	6	90.51 ± 24.73	>0.9999	0.29 [-0.7; 1.3]
		**ZEM 25**	6	82.35 ± 4.114	>0.9999	-0.2 [-1.2; 0.8]
		**MES**	6	76.31 ± 11.56	>0.9999	-0.6 [-1.6; 0.4]
**ELEVATED PLUS MAZE (EPM)**						
**Distance (cm)**	**X²(4) = 8.3, *p* = 0.0813***	**UCMS**	12	1832 ± 268.6		
		**ESC**	6	1680 ± 142.5	0.9927	-0.61 [-1.6; 0.4]
		**ZEM 12.5**	6	1730 ± 162.7	>0.9999	-0.4 [-1.4; 0.6]
		**ZEM 25**	6	1739 ± 182.5	>0.9999	-0.36 [-1.4; 0.6]
		**MES**	6	1997 ± 114.9	0.2954	0.68 [-0.3; 1.7]
**Open/closed arm duration**	X²(4) = 3.42, *p* = 0.4907	**UCMS**	12	1.555 ± 0.4705		
		**ESC**	6	1.322 ± 0.5313	>0.9999	-0.45 [-1.5; 0.5]
		**ZEM 12.5**	6	2.017 ± 0.9687	>0.9999	0.66 [-0.3; 1.7]
		**ZEM 25**	6	1.358 ± 0.6794	>0.9999	-0.35 [-1.3; 0.6]
		**MES**	6	1.564 ± 0.5214	>0.9999	0.02 [-1.0; 1.0]
**BARNES MAZE (BM)**						
**Distance (s)**	X²(4) = 7.24, *p* = 0.1238	**UCMS**	12	1343 ± 393.6		
		**ESC**	6	1208 ± 184.1	>0.9999	-0.37 [-1.4; 0.6]
		**ZEM 12.5**	6	1156 ± 219.1	>0.9999	-0.51 [-1.5; 0.5]
		**ZEM 25**	6	1317 ± 401.1	>0.9999	-0.06 [-1.0; 0.9]
		**MES**	6	1790 ± 407.7	0.1651	**1.07 [0.1; 2.2]**
**Primary latency (s)**	X²(4) = 2.448, *p* = 0.654	**UCMS**	12	29.09 ± 28.48		
		**ESC**	6	13.17 ± 10.07	0.9911	-0.63 [-1.7; 0.4]

		ZEM 12.5	6	20.75 ± 20.01	>0.9999	-0.29 [-1.5; 0.8]
		ZEM 25	6	28.50 ± 17.99	>0.9999	-0.02 [-1.0; 1.0]
		MES	6	28.00 ± 27.87	>0.9999	-0.04 [-1.0; 1.0]
**Primary error rate**	$X^2(4) = 2.335$, $p = 0.6744$	UCMS	12	7.600 ± 6.931		
		ESC	6	4.667 ± 3.882	>0.9999	-0.46 [-1.5; 0.5]
		ZEM 12.5	6	7.250 ± 8.139	>0.9999	-0.05 [-1.2; 1.1]
		ZEM 25	6	11.17 ± 9.174	>0.9999	0.43 [-0.6; 1.5]
		MES	6	10.50 ± 10.54	>0.9999	0.33 [-0.7; 1.4]
**Edge exploration (head dips)**	**$X^2(4) = 9.23$, $p = 0.0556$***	UCMS	12	10.17 ± 3.099		
		ESC	6	9.167 ± 2.563	>0.9999	-0.32 [-1.3; 0.7]
		ZEM 12.5	6	10.83 ± 2.137	>0.9999	0.22 [-0.8; 1.2]
		ZEM 25	6	12.00 ± 3.688	0.8312	0.53 [-0.5; 1.5]
		MES	6	15.33 ± 4.082	**0.0449**	**1.43 [0.4; 2.6]**
**Probe/Not probe duration**	$X^2(4) = 5.52$, $p = 0.2380$	UCMS	12	0.48 ± 0.37		
		ESC	6	0.87 ± 0.57	0.5476	0.84 [-0.2; 1.9]
		ZEM 12.5	6	0.35 ± 0.23	>0.9999	-0.38 [-1.4; 0.6]
		ZEM 25	6	0.64 ± 0.31	0.7352	0.43 [-0.6; 1.4]
		MES	6	0.35 ± 0.09	>0.9999	-0.4 [-1.4; 0.6]
**Outer/inner duration**	$X^2(4) = 1.06$, $p = 0.9011$	UCMS	12	89.29 ± 152.2		
		ESC	6	101.5 ± 134.2	>0.9999	0.08 [-0.9; 1.1]
		ZEM 12.5	6	54.76 ± 57.88	>0.9999	-0.25 [-1.2; 0.7]
		ZEM 25	6	27.51 ± 27.43	>0.9999	-0.46 [-1.5; 0.5]
		MES	6	33.03 ± 29.31	>0.9999	-0.42 [-1.4; 0.6]

Table S2. Summary of results for the neurochemical analyses in the frontal cortex and hippocampus presented for each treatment (Rx) group for females alone. $p \leq 0.1$ Outliers indicated as n^O (outlier not removed) or n^R (outlier removed). 3-CLT – 3-chlorotyrosine; 3-CLT/TYR – 3-CLT turnover rate; 5-HIAA – 5-Hydroxyindoleacetic acid; 5-HT – serotonin; 5-HIAA/5-HT – serotonin turnover rate ; 95% CI – 95% confidence intervals; DA –- dopamine; DOPAC – 3.4-dihydroxyphenylacetic acid; DOPAC/DA – dopamine turnover rate; ESC – Escitalopram; GSH – reduced glutathione; GSSG – oxidised glutathione; GSSG/GSH – GSH turnover; MES – Mesembrine; NA – noradrenaline; PDE4B – phosphodiesterase 4B; PND – postnatal day; SD – standard deviation; UCMS – saline-treated stress group; TYR – tyrosine; ZEM 12.5 and 25 - Zembrin® 12.5 and 25 mg/kg.

Marker	Kruskal-Wallis test	Rx Group	n	Mean ± SD	*p*-value	*d* [95 % CI]
**FRONTAL CORTEX**						
**PDE4B (ng/ml)**	**$X^2(4) = 14.54$, $p = 0.0058$****	UCMS	10	1.22 ± 0.17		
		ESC	5	1.49 ± 0.21	0.2724	1.41 [0.3; 2.7]
		ZEM 12.5	5	0.87 ± 0.10	**0.0391**	**-2.19 [-3.7; -0.9]**
		ZEM 25	5	1.16 ± 0.12	>0.9999	-0.39 [-1.5; 0.7]
		MES	5	1.18 ± 0.14	>0.9999	-0.21 [-1.3; 0.9]
**5-HT (ng/g wet brain)**	$X^2(4) = 1.31$, $p = 0.8593$	UCMS	12	127.80 ± 40.66		
		ESC	6	134.61 ± 64.94	>0.9999	0.13 [-0.9; 1.1]
		ZEM 12.5	6	143.40 ± 29.15	>0.9999	0.4 [-0.6; 1.4]

		**ZEM 25**	6	129.10 ± 20.70	>0.9999	0.04 [-0.9; 1.0]
		**MES**	6	138.21 ± 40.96	>0.9999	0.24 [-0.7; 1.2]
**5-HIAA (ng/g wet brain)**	**$X^2(4) = 10.11$, $p = 0.0386$***	**UCMS**	12○	206.60 ± 53.59		
		**ESC**	6	231.80 ± 22.84	0.8928	0.52 [-0.5; 1.5]
		**ZEM 12.5**	6	278.31 ± 37.83	**0.0112**	**1.39 [0.3; 2.5]**
		**ZEM 25**	6○	221.81 ± 27.93	>0.9999	0.31 [-0.7; 1.3]
		**MES**	6	202.40 ± 53.28	>0.9999	-0.07 [-1.1; 0.9]
**5-HIAA/5-HT (ng/g wet brain)**	$X^2(4) = 6.89$, $p = 0.1417$	**UCMS**	12	1.16 ± 0.70		
		**ESC**	6	2.00 ± 0.73	0.1258	**1.12 [0.1; 2.2]**
		**ZEM 12.5**	6	2.05 ± 0.68	0.1471	**1.23 [0.2; 2.4]**
		**ZEM 25**	6	1.77 ± 0.44	0.6556	0.92 [-0.1; 2.0]
		**MES**	6	1.60 ± 0.67	>0.9999	0.61 [-0.4; 1.6]
**DA (ng/g wet brain)**	$X^2(4) = 9.23$, $p = 0.0557$	**UCMS**	12	179.3 ± 130.2		
		**ESC**	6	131.2 ± 49.08	>0.9999	-0.41 [-1.4; 0.6]
		**ZEM 12.5**	6	166.0 ± 61.38	>0.9999	-0.11 [-1.1; 0.9]
		**ZEM 25**	6	216.9 ± 39.49	0.3502	0.33 [-0.7; 1.3]
		**MES**	6	100.9 ± 41.21	0.3997	-0.68 [-1.7; 0.3]
**DOPAC (ng/g wet brain)**	**$X^2(4) = 13.21$, $p = 0.0103$***	**UCMS**	12	94.60 ± 35.93		
		**ESC**	6	155.7 ± 59.00	**0.0077**	**1.31 [0.3; 2.5]**
		**ZEM 12.5**	6	121.3 ± 20.56	0.1715	0.8 [-0.2; 1.9]
		**ZEM 25**	6	95.01 ± 11.00	>0.9999	0.01 [-1.0; 1.0]
		**MES**	6	121.2 ± 38.84	0.6181	0.69 [-0.3; 1.7]
**DOPAC/DA**	**$X^2(4) = 19.95$, $p = 0.0005$*****	**UCMS**	12	0.72 ± 0.37		
		**ESC**	6	1.19 ± 0.19	**0.0415**	**1.38 [0.3; 2.5]**
		**ZEM 12.5**	6	0.77 ± 0.16	>0.9999	0.16 [-0.8; 1.1]
		**ZEM 25**	6	0.44 ± 0.05	0.5823	-0.86 [-1.9; 0.1]
		**MES**	6	1.25 ± 0.22	**0.0260**	**1.5 [0.4; 2.7]**
**NA (ng/g wet brain)**	**$X^2(4) = 8.83$, $p = 0.0655$**	**UCMS**	12	4574 ± 2270		
		**ESC**	6	6736 ± 2557	0.1920	0.87 [-0.1; 1.9]
		**ZEM 12.5**	6	3333 ± 1193	>0.9999	-0.59 [-1.6; 0.4]
		**ZEM 25**	6	5457 ± 1354	0.7567	0.42 [-0.6; 1.4]
		**MES**	6	6196 ± 3254	0.7567	0.59 [-0.4; 1.6]
**Tyrosine (TYR) (ng/g wet brain)**	$X^2(4) = 5.23$, $p = 0.2645$	**UCMS**	12	109.1 ± 47.88		
		**ESC**	6	140.0 ± 39.28	0.2568	0.65 [-0.3; 1.7]
		**ZEM 12.5**	6○	87.88 ± 42.60	>0.9999	-0.44 [-1.4; 0.5]
		**ZEM 25**	6	115.4 ± 18.07	>0.9999	0.15 [-0.8; 1.1]
		**MES**	6	128.0 ± 49.26	>0.9999	0.37 [-0.6; 1.4]
**3-CLT (ng/g wet brain)**	**$X^2(4) = 7.96$, $p = 0.0931$**	**UCMS**	12	96.36 ± 38.93		
		**ESC**	6	124.4 ± 35.37	0.2144	0.7 [-0.28; 1.7]
		**ZEM 12.5**	6○	74.63 ± 35.74	>0.9999	-0.55 [-1.6; 0.4]
		**ZEM 25**	6	112.0 ± 15.28	0.4265	0.45 [-0.5; 1.5]

		**MES**	6	120.4 ± 45.06	0.8690	0.56 [-0.4; 1.6]
**3-CLT/TYR**	**$X^2(4) = 13.64$, $p = 0.0086$****	**UCMS**	12	0.9 ± 0.08		
		**ESC**	6	0.89 ± 0.01	>0.9999	-0.15 [-1.1; 0.8]
		**ZEM 12.5**	6	0.86 ± 0.03	0.9418	-0.58 [-1.6; 0.4]
		**ZEM 25**	6	0.97 ± 0.04	**0.0568**	**1.1 [0.1; 2.2]**
		**MES**	6	0.94 ± 0.04	0.4129	0.67 [-0.3; 1.71]
**GSH**<br>(ng/g wet brain)	**$X^2(4) = 13.60$, $p = 0.0087$****	**UCMS**	12°	76.60 ± 19.25		
		**ESC**	6	89.77 ± 14.07	0.8456	0.71 [-0.3; 1.7]
		**ZEM 12.5**	6°	66.62 ± 30.07	>0.9999	-0.41 [-1.4; 0.6]
		**ZEM 25**	6	108.1 ± 7.503	**0.0047**	**1.82 [0.7; 3.1]**
		**MES**	6	85.80 ± 15.92	>0.9999	0.48 [-0.5; 1.5]
**GSSG**<br>(ng/g wet brain)	$X^2(4) = 6.63$, $p = 0.1566$	**UCMS**	12	160.7 ± 98.64		
		**ESC**	6	129.7 ± 11.38	>0.9999	0.71 [-0.3; 1.7]
		**ZEM 12.5**	6°	124.5 ± 56.30	>0.9999	-0.41 [-1.4; 0.6]
		**ZEM 25**	6	184.4 ± 20.34	0.2954	1.82 [0.7; 3.1]
		**MES**	6°	124.0 ± 45.17	>0.9999	0.48 [-0.5; 1.5]
**GSSG/GSH**<br>(ng/g wet brain)	$X^2(4) = 3.99$, $p = 0.4080$	**UCMS**	12	2.127 ± 1.210		
		**ESC**	6	1.462 ± 0.1574	>0.9999	-0.63 [-1.7; 0.4]
		**ZEM 12.5**	6	1.832 ± 0.2578	>0.9999	-0.28 [-1.3; 0.7]
		**ZEM 25**	6	1.712 ± 0.2253	>0.9999	-0.39 [-1.4; 0.6]
		**MES**	6	1.469 ± 0.5465	0.8690	-0.60 [-1.6; 0.4]
**HIPPOCAMPUS**						
**PDE4B**<br>**(ng/ml)**	**$X^2(4) = 12.02$, $p = 0.172$***	**UCMS**	10	1.19 ± 0.19		
		**ESC**	5°	1.09 ± 0.12	>0.9999	-0.55 [-1.7; 0.5]
		**ZEM 12.5**	5	0.93 ± 0.10	**0.0189**	**-1.46 [-2.8; -0.3]**
		**ZEM 25**	5	1.12 ± 0.14	>0.9999	-0.38 [-1.5; 0.7]
		**MES**	5	0.96 ± 0.07	**0.0757**	**-1.35 [-2.6; -0.2]**
**5-HT**<br>**(ng/g wet brain)**	$X^2(4) = 4.132$, $p = 0.3884$	**UCMS**	12°	121.5 ± 107.7		
		**ESC**	6	83.62 ± 26.55	>0.9999	-0.4 [-1.4; 0.6]
		**ZEM 12.5**	6°	162.0 ± 156.3	0.6556	0.31 [-0.7; 1.3]
		**ZEM 25**	6°	208.7 ± 201.6	0.8227	0.58 [-0.4; 1.6]
		**MES**	6°	125.4 ± 121.9	>0.9999	0.03 [-1.0; 1.0]
**5-HIAA**<br>**(ng/g wet brain)**	**$X^2(4) = 10.35$, $p = 0.0350$***	**UCMS**	12	286.1 ± 70.06		
		**ESC**	6	250.2 ± 41.20	0.9418	-0.55 [-1.6; 0.4]
		**ZEM 12.5**	6	375.2 ± 96.16	0.1209	**1.07 [0.1; 2.2]**
		**ZEM 25**	6	322.9 ± 125.1	0.6749	0.39 [-0.6; 1.4]
		**MES**	6	301.1 ± 34.56	>0.9999	0.23 [-0.7; 1.2]
**5-HIAA/5-HT**	$X^2(4) = 4.00$, $p = 0.4065$	**UCMS**	12	3.14 ± 1.199		
		**ESC**	6°	3.34 ± 1.58	>0.9999	0.15 [-0.8; 1.1]
		**ZEM 12.5**	6	3.25 ± 1.67	>0.9999	0.08 [-0.9; 1.1]
		**ZEM 25**	6	2.30 ± 1.39	0.3868	-0.636 [-1.7; 0.4]

		**MES**	6	3.42 ± 1.35	>0.9999	0.22 [-0.8; 1.2]
**DA** (ng/g wet brain)	$X^2(4) = 4.79$, $p = 0.3100$	**UCMS**	12°	67.48 ± 34.49		
		**ESC**	6	62.78 ± 12.78	>0.9999	-0.15 [-1.1; 0.8]
		**ZEM 12.5**	6°	68.85 ± 25.86	>0.9999	0.04 [-0.9; 1.0]
		**ZEM 25**	6	48.06 ± 17.85	>0.9999	-0.61 [-1.6; 0.4]
		**MES**	6	51.16 ± 6.73	0.7567	-0.54 [-1.6; 0.4]
**DOPAC** (ng/g wet brain)	**$X^2(4) = 9.38$, $p = 0.0523$**	**UCMS**	12°	83.48 ± 35.49		
		**ESC**	6	79.90 ± 15.80	>0.9999	-0.11 [-1.1; 0.9]
		**ZEM 12.5**	6	81.32 ± 18.63	>0.9999	-0.07 [-1.1; 0.9]
		**ZEM 25**	6	52.76 ± 14.75	**0.0544**	-0.96 [-2.0; 0.1]
		**MES**	6°	67.14 ± 11.80	>0.9999	-0.52 [-1.5; 0.5]
**DOPAC/DA**	$X^2(4) = 1.847$, $p = 0.7639$	**UCMS**	12	1.28 ± 0.20		
		**ESC**	6	1.28 ± 0.17	>0.9999	0.03 [-1.0; 1.0]
		**ZEM 12.5**	6	1.30 ± 0.50	>0.9999	0.08 [-0.9; 1.1]
		**ZEM 25**	6°	1.16 ± 0.27	>0.9999	-0.49 [-1.5; 0.5]
		**MES**	6	1.32 ± 0.20	>0.9999	0.19 [-0.8; 1.2]
**NA** (ng/g wet brain)	**$X^2(4) = 9.658$, $p = 0.0466$***	**UCMS**	12	3687 ± 2469		
		**ESC**	6	3158 ± 816.9	>0.9999	-0.24 [-1.2; 0.7]
		**ZEM 12.5**	6	3332 ± 1250	>0.9999	-0.16 [-1.1; 0.8]
		**ZEM 25**	6	9692 ± 10071	>0.9999	0.95 [-0.1; 2.0]
		**MES**	6	1645 ± 635.6	0.1071	-0.94 [-2.0; 0.1]
**Tyrosine (TYR)** (ng/g wet brain)	$X^2(4) = 3.28$, $p = 0.5122$	**UCMS**	12°	137.6 ± 63.28		
		**ESC**	6	111.4 ± 19.34	>0.9999	-0.47 [-1.5; 0.5]
		**ZEM 12.5**	6	130.3 ± 34.06	>0.9999	-0.12 [-1.1; 0.9]
		**ZEM 25**	6	99.99 ± 18.21	0.6556	-0.67 [-1.7; 0.3]
		**MES**	6	104.3 ± 17.86	>0.9999	0.00 [-0.8; 0.8]
**3-CLT** (ng/g wet brain) *Some below limit of detection*	$X^2(4) = 0.98$, $p = 0.9128$	**UCMS**	*9*^{O}	116.1 ± 60.46		
		**ESC**	*5*	91.40 ± 18.52	>0.9999	-0.46 [-1.6; 0.6]
		**ZEM 12.5**	*3*	82.41 ± 6.865	>0.9999	-0.57 [-1.9; 0.7]
		**ZEM 25**	*6*	90.98 ± 18.22	>0.9999	-0.49 [-1.6; 0.5]
		**MES**	*6*	91.88 ± 14.08	>0.9999	-0.47 [-1.5; 0.6]
**3-CLT/TYR** (ng/g wet brain) *Some below limit of detection*	**$X^2(4) = 13.03$, $p = 0.0111$***	**UCMS**	*9*	0.84 ± 0.04		
		**ESC**	*5*	0.83 ± 0.02	>0.9999	-0.19 [-1.3; 0.9]
		**ZEM 12.5**	*3*	0.83 ± 0.01	>0.9999	-0.27 [-1.6; 1.0]
		**ZEM 25**	*6*	0.91 ± 0.03	**0.0323**	**1.73 [0.6; 3.1]**
		**MES**	*6*	0.88 ± 0.05	0.3986	0.98 [-0.1; 2.1]
**GSH** (ng/g wet brain)	**$X^2(4) = 12.56$, $p = 0.0136$****	**UCMS**	12	91.40 ± 16.05		
		**ESC**	6	90.42 ± 12.80	>0.9999	-0.06 [-1.0; 0.9]
		**ZEM 12.5**	6	70.17 ± 5.922	**0.0106**	**-1.5 [-2.6; -0.4]**
		**ZEM 25**	6	94.35 ± 14.44	>0.9999	0.18 [-0.8; 1.2]
		**MES**	6	83.53 ± 8.813	>0.9999	-0.53 [-1.5; 0.5]

**GSSG (ng/g wet brain)**	**$X^2(4) = 14.01$, $p = 0.0073$****	**UCMS**	12	216.0 ± 87.39		
		**ESC**	5[R]	134.4 ± 23.40	0.1130	-1.07 [-2.2; 0.0]
		**ZEM 12.5**	6	122.7 ± 20.67	**0.0166**	**-1.22 [-2.4; -0.2]**
		**ZEM 25**	6	179.3 ± 18.84	>0.9999	-0.48 [-1.5; 0.5]
		**MES**	6	150.8 ± 8.693	>0.9999	-0.87 [-1.9; 0.1]
**GSSG/GSH**	**$X^2(4) = 13.99$, $p = 0.0073$****	**UCMS**	12	2.68 ± 1.19		
		**ESC**	6	1.48 ± 0.07	**0.0020**	**-1.16 [-2.3; -0.1]**
		**ZEM 12.5**	6	1.75 ± 0.21	0.9670	-0.89 [-2.0; 0.1]
		**ZEM 25**	6	1.92 ± 0.23	>0.9999	-0.72 [-1.8; 0.3]
		**MES**	6	1.82 ± 0.16	>0.9999	-0.83 [-1.9; 0.2]
**PLASMA**						
**TNF-α (pg/ml)**	$X^2(4) = 5.83$, $p = 0.2122$	**UCMS**	12	7.463 ± 3.441		
		**ESC**	6	4.502 ± 3.503	0.9405	-0.81 [-2.0; 0.3]
		**ZEM 12.5**	6	9.274 ± 1.215	>0.9999	0.58 [-0.5; 1.7]
		**ZEM 25**	6	9.986 ± 2.139	>0.9999	0.0 [-0.9; 0.9]
		**MES**	6	9.800 ± 6.149	>0.9999	0.49 [-0.6; 1.6]
**IL-10 (pg/ml)**	$X^2(4) = 4.56$, $p = 0.3354$	**UCMS**	12	16.95 ± 7.287		
		**ESC**	6	15.29± 9.907	>0.9999	-0.19 [-1.3; 0.9]
		**ZEM 12.5**	6	22.90 ± 19.66	>0.9999	0.45 [-0.6; 1.6]
		**ZEM 25**	6	10.88 ± 1.491	0.5380	-0.93 [-2.1; 0.2]
		**MES**	6	10.34 ± 1.705	0.2456	-1.01 [-2.2; 0.1]

Table S3. Summary of results for the behavioural tests presented for each treatment (Rx) group for both sexes combined. $p \leq 0.05$. Outliers indicated as n[O] (outlier not removed) or n[R] (outlier removed). PND – Postnatal day; SD – standard deviation; 95% CI – 95% confidence intervals; UCMS – Saline-treated stress group; ESC – Escitalopram; ZEM 12.5 and 25 - Zembrin®; MES – Mesembrine.

Parameter	Kruskal-Wallis test	Rx Group	n	Mean ± SD	*p*-value	*d* [95 % CI]
**SUCROSE PREFERENCE TEST (SPT)**						
**Sucrose preference PND 50**	$X^2(4) = 3.76$, $p = 0.4391$	**UCMS**	24[O]	0.69 ± 0.23		
		**ESC**	11[O]	0.64 ± 0.19	0.8252	0.26 [-0.5; 1.0]
		**ZEM 12.5**	12[O]	0.63 ± 0.18	0.5086	-0.45 [-0.3; 1.2]
		**ZEM 25**	12[O]	0.70 ± 0.22	>0.9999	0.5 [-0.2; 1.2]
		**MES**	12	0.68 ± 0.24	>0.9999	-0.36 [-1.1; 0.3]
**Sucrose preference PND 64**	$X^2(4) = 7.99$, $p = 0.0917$	**UCMS**	24	0.74 ± 0.19		
		**ESC**	11[O]	0.84 ± 0.14	0.2040	0.53 [-0.2; 1.3]
		**ZEM 12.5**	12[O]	0.75 ± 0.16	>0.9999	0.05 [-0.6; 0.8]
		**ZEM 25**	12[O]	0.75 ± 0.18	>0.9999	0.04 [-0.7; 0.7]
		**MES**	12	0.85 ± 0.05	0.6759	0.66 [0.0; 1.4]

**Sucrose preference PND 78**	$X^2(4) = 7.77$, $p = 0.1003$	**UCMS**	24 ○	0.77 ± 0.15		
		**ESC**	11	0.81 ± 0.17	0.3619	0.26 [-0.5; 1.0]
		**ZEM 12.5**	12	0.83 ± 0.06	>0.9999	0.45 [-0.3; 1.2]
		**ZEM 25**	12 ○	0.84 ± 0.04	>0.9999	0.5 [-0.2; 1.2]
		**MES**	12	0.87 ± 0.06	**0.0338**	**0.75 [0.0; 1.5]**
**Sucrose preference PND 92**	$X^2(4) = 8.38$, $p = 0.0785$	**UCMS**	24	0.73 ± 0.23		
		**ESC**	11 ○	0.83 ± 0.20	**0.0227**	0.45 [-0.3; 1.2]
		**ZEM 12.5**	12	0.81 ± 0.12	>0.9999	0.39 [-0.3; 1.1]
		**ZEM 25**	12	0.82 ± 0.10	0.9986	0.46 [-0.2; 1.2]
		**MES**	12	0.79 ± 0.12	>0.9999	0.31 [-0.4; 1.0]
**Sucrose preference PND 101**	**$X^2(4) = 13.14$, $p = 0.0106$***	**UCMS**	24	0.72 ± 0.27		
		**ESC**	11	0.80 ± 0.10	>0.9999	0.33 [-0.4; 1.1]
		**ZEM 12.5**	12	0.90 ± 0.03	**0.0274**	**0.78 [0.1; 1.5]**
		**ZEM 25**	12	0.85 ± 0.07	>0.9999	0.57 [-0.1; 1.3]
		**MES**	12 ○	0.76 ± 0.17	>0.9999	0.14 [-0.6; 0.8]
**FORCED SWIM TEST (FST)**						
**Swimming (s)**	**$X^2(4) = 21.72$, $p = 0.0002$*****	**UCMS**	24	91.47 ± 46.33		
		**ESC**	11	67.76 ± 38.61	0.4490	-0.53 [-1.3; 0.2]
		**ZEM 12.5**	12	40.08 ± 23.77	**0.0020**	**-1.24 [-2.0; -0.5]**
		**ZEM 25**	12	111.7 ± 43.62	0.6894	0.44 [-0.3; 1.1]
		**MES**	12	90.24 ± 29.29	>0.9999	-0.03 [-0.7; 0.7]
**Immobility (s)**	**$X^2(4) = 20.91$, $p = 0.0003$*****	**UCMS**	24	196.3 ± 46.28		
		**ESC**	11	223.8 ± 39.14	0.3296	0.61 [-0.1; 1.3]
		**ZEM 12.5**	12	251.4 ± 24.89	**0.0023**	**1.33 [0.6; 2.1]**
		**ZEM 25**	12	178.5 ± 41.87	>0.9999	-0.39 [-1.1; 0.3]
		**MES**	12	190.4 ± 34.42	>0.9999	-0.14 [-0.8; 0.6]
**Struggling (s)**	$X^2(4) = 7.51$, $p = 0.1112$	**UCMS**	24	12.30 ± 12.71		
		**ESC**	11	7.99 ± 10.56	0.8465	-0.35 [-1.1; 0.4]
		**ZEM 12.5**	12	8.67 ± 10.98	>0.9999	-0.29 [-1.0; 0.4]
		**ZEM 25**	12	9.74 ± 8.07	>0.9999	-0.22 [-0.9; 0.5]
		**MES**	12	19.62 ± 15.85	0.4864	0.52 [-0.2; 1.2]
**OPEN FIELD TEST (OFT)**						
**Distance moved (cm)**	$X^2(4) = 8.69$, $p = 0.0692$	**UCMS**	24	9549 ± 1235		
		**ESC**	11	8862 ± 1046	0.4052	-0.57 [-1.0; 0.2]
		**ZEM 12.5**	12 ○	8263 ± 1230	**0.0225**	**-1.02 [-1.8; -0.3]**
		**ZEM 25**	12	9453 ± 1273	>0.9999	-0.08 [-0.8; 0.6]
		**MES**	12	9069 ± 1403	>0.9999	-0.36 [-1.1; 0.3]
**Center duration (s)**	**$X^2(4) = 8.69$, $p = 0.0024$****	**UCMS**	24	62.58 ± 14.71		
		**ESC**	11	44.26 ± 8.956	**0.0090**	**-1.35 [-2.2; -0.6]**
		**ZEM 12.5**	12	51.00 ± 25.13	0.2290	-0.61 [-1.3; 0.1]
		**ZEM 25**	12	68.43 ± 22.29	>0.9999	0.33 [-0.4; 1.0]

		**MES**	12	63.48 ± 12.32	>0.9999	0.06 [-0.6; 0.8]
**Corner duration (s)**	$X^2(4) = 8.69$, $p = 0.0560$	**UCMS**	24	73.27 ± 16.60		
		**ESC**	11	94.10 ± 19.56	**0.0117**	**1.16 [0.4; 1.9]**
		**ZEM 12.5**	12	82.62 ± 24.86	>0.9999	0.47 [-0.2; 1.2]
		**ZEM 25**	12	76.04 ±16.94	>0.9999	0.37 [-0.3; 1.1]
		**MES**	12	75.12 ± 16.69	>0.9999	0.11 [-0.6; 0.8]
**ELEVATED PLUS MAZE (EPM)**						
**Distance (cm)**	$X^2(4) = 3.68$, $p = 0.4506$	**UCMS**	24	1797 ± 235.1		
		**ESC**	11	1780 ±231.4	>0.9999	-0.07 [-0.8; 0.6]
		**ZEM 12.5**	12	1755 ± 276.4	>0.9999	-0.17 [-0.9; 0.5]
		**ZEM 25**	12	1823 ± 308.3	>0.9999	0.1 [-0.6; 0.8]
		**MES**	12	1907 ± 143.4	0.4708	0.51 [-0.2; 1.2]
**Open/closed arm duration**	$X^2(4) = 6.37$, $p = 0.1735$	**UCMS**	24	1.587 ± 0.56		
		**ESC**	11	1.298 ± 0.55	0.9805	-0.51 [-1.2; 0.2]
		**ZEM 12.5**	12	2.185 ± 0.96	0.3304	0.82 [0.1; 1.6]
		**ZEM 25**	12	1.797 ± 1.19	>0.9999	0.25 [-0.4; 1.0]
		**MES**	12	1.630 ± 0.47	>0.9999	0.08 [-0.6; 0.8]
**BARNES MAZE (BM)**						
**Distance (s)**	**$X^2(4) = 11.90$, $p = 0.0181$***	**UCMS**	24	1321 ± 295.7		
		**ESC**	11	1165 ± 205.1	0.8682	-0.56 [-1.3; 0.2]
		**ZEM 12.5**	12	1073 ± 202.3	0.0712	-0.9 [-1.6; -0.2]
		**ZEM 25**	12	1356 ± 285.0	>0.9999	0.12 [-0.6; 0.8]
		**MES**	12	1528 ± 418.8	0.9671	0.6 [-0.1; 1.3]
**Primary latency (s)**	$X^2(4) = 1.18$, $p = 0.8814$	**UCMS**	24	23.33 ± 22.98		
		**ESC**	11	20.40 ± 14.60	>0.9999	-0.14 [-0.9; 0.6]
		**ZEM 12.5**	12	27.67 ± 22.43	>0.9999	0.19 [-0.6; 1.0]
		**ZEM 25**	12	25.50 ± 17.33	>0.9999	0.1 [-0.6; 0.8]
		**MES**	12	29.00 ± 29.32	>0.9999	0.22 [-0.5; 1.0]
**Primary error rate**	$X^2(4) = 1.18$, $p = 0.8814$	**UCMS**	24	5.842 ± 5.728		
		**ESC**	11	7.300 ± 5.314	>0.9999	0.25 [-0.5; 1.0]
		**ZEM 12.5**	12	9.444 ± 8.487	>0.9999	0.52 [-0.3; 1.3]
		**ZEM 25**	12	8.167 ± 7.284	>0.9999	0.36 [-0.4; 1.1]
		**MES**	12	8.545 ± 8.513	>0.9999	0.38 [-0.4; 1.1]
**Edge exploration (head dips)**	**$X^2(4) = 11.03$, $p = 0.0262$***	**UCMS**	24	11.38 ± 3.704		
		**ESC**	11	8.545 ± 2.296	0.1168	-0.83 [-1.6; -0.1]
		**ZEM 12.5**	12	10.58 ± 2.575	>0.9999	-0.23 [-0.9; 0.5]
		**ZEM 25**	12	11.17 ± 3.186	>0.9999	-0.06 [-0.8; 0.6]
		**MES**	12	13.50 ± 4.056	0.4237	0.54 [-0.2; 1.3]
**Probe/Not probe duration**	$X^2(4) = 1.87$, $p = 0.7596$	**UCMS**	24	0.59 ± 0.43		
		**ESC**	11	0.73 ± 0.51	>0.9999	0.29 [-0.4; 1.0]
		**ZEM 12.5**	12	0.56 ± 0.40	>0.9999	-0.07 [-0.8; 0.6]

		**ZEM 25**	12	0.58 ± 0.31	>0.9999	-0.03 [-0.7; 0.7]
		**MES**	12	0.43 ± 0.21	>0.9999	-0.44 [-1.1; 0.3]
**Outer/inner duration**	$X^2(4) = 1.31$, *p* = 0.8594	**UCMS**	24	28.43 ± 42.58		
		**ESC**	11	60.16 ±106.4	>0.9999	0.45 [-0.3; 1.2]
		**ZEM 12.5**	12	40.34 ± 46.18	>0.9999	0.27 [-0.4; 1.0]
		**ZEM 25**	12	19.23 ± 21.99	>0.9999	-0.24 [-0.9; 0.5]
		**MES**	12	33.01 ± 33.28	>0.9999	0.11 [-0.6; 0.8]

Table S4. Summary of results for the neurochemical analyses in the frontal cortex and hippocampus presented for each treatment (Rx) group for both sexes combined. p < 0.05. Outliers indicated as n^O (outlier not removed) or n^R (outlier removed). 3-CLT – 3-chlorotyrosine; 3-CLT/TYR – 3-CLT turnover rate; 5-HIAA – 5-Hydroxyindoleacetic acid; 5-HT – serotonin; 5-HIAA/5-HT – serotonin turnover rate ; 95% CI – 95% confidence intervals; DA –- dopamine; DOPAC – 3.4-dihydroxyphenylacetic acid; DOPAC/DA – dopamine turnover rate; ESC – Escitalopram; GSH – reduced glutathione; GSSG – oxidised glutathione; GSSG/GSH – GSH turnover; MES – Mesembrine; NA – noradrenaline; PDE4B – phosphodiesterase 4B; PND – postnatal day; SD – standard deviation; UCMS – saline-treated stress group; TYR – tyrosine; ZEM 12.5 and 25 - Zembrin® 12.5 and 25 mg/kg.

Marker	Kruskal-Wallis test	Rx Group	n	Mean ± SD	*p*-value	*d* [95 % CI]
**FRONTAL CORTEX**						
**PDE4B (ng/ml)**	$X^2(4) = 25.14$, *p* = <0.0001****	**UCMS**	20	1.2 ± 0.2213		
		**ESC**	10	1.38 ± 0.2348	>0.9999	0.4 [-0.4; 1.2]
		**ZEM 12.5**	10°	0.86 ± 0.07314	**<0.0001********	**-2.24 [-3.3; -1.3]**
		**ZEM 25**	10	1.16 ± 0.1125	0.8044	-0.64 [-1.4; 0.1]
		**MES**	10	1.25 ± 0.1544	>0.9999	-0.2 [-1.0; 0.6]
**5-HT (ng/g wet brain)**	$X^2(4) = 1.61$, *p* = 0.8070	**UCMS**	24	119.60 ± 37.24		
		**ESC**	11	137.30 ± 50.12	>0.9999	0.42 [-0.3; 1.1]
		**ZEM 12.5**	12	129.91 ± 32.34	>0.9999	0.28 [-0.4; 1.0]
		**ZEM 25**	12°	125.30 ± 36.15	>0.9999	0.15 [-0.5; 0.9]
		**MES**	12°	146.21 ± 104.3	>0.9999	0.39 [-0.3; 1.1]
**5-HIAA (ng/g wet brain)**	**$X^2(4) = 23.19$, *p* = 0.0001*** **	**UCMS**	24	215.70 ± 55.44		
		**ESC**	11	277.31 ± 66.21	0.0574	1.02 [0.3; 1.8]
		**ZEM 12.5**	12	276.70 ± 33.86	**0.0053**	**1.21 [0.5; 2.0]**
		**ZEM 25**	12	209.80 ± 29.31	>0.9999	-0.12 [-0.8; 0.6]
		**MES**	12	198.21 ± 42.65	>0.9999	-0.33 [-1.0; 0.4]
**5-HIAA/5-HT**	**$X^2(4) = 10.65$, *p* = 0.0308***	**UCMS**	24	1.54 ± 0.8197		
		**ESC**	11	2.18 ± 0.5974	0.0555	1.02 [0.3; 1.8]
		**ZEM 12.5**	12	2.26 ± 0.5953	**0.0386**	**1.21 [0.5; 2.0]**
		**ZEM 25**	12	1.77 ± 0.4463	>0.9999	-0.12 [-0.8; 0.6]
		**MES**	12	1.80 ± 0.5330	>0.9999	-0.33 [-1.0; 0.4]
**DA**	**$X^2(4) = 16.84$, *p* = 0.0021****	**UCMS**	24	216.00 ± 162.4		
		**ESC**	11°	155.20 ± 90.69	0.7441	-0.41 [-1.1; 0.3]

**(ng/g wet brain)**		**ZEM 12.5**	12[O]	194.51 ± 103.1	>0.9999	-0.14 [-0.8; 0.6]
		**ZEM 25**	12	226.00 ± 63.80	0.3503	0.07 [-0.6; 0.8]
		**MES**	11[R]	111.40 ± 44.63	**0.0280**	**-0.75 [-1.5; -0.1]**
**DOPAC (ng/g wet brain)**	$X^2(4) = 6.68, p = 0.1536$	**UCMS**	24[O]	123.60 ± 64.45		
		**ESC**	11	149.61 ± 55.75	0.2821	0.41 [-0.3; 1.1]
		**ZEM 12.5**	12[O]	146.40 ± 85.27	0.9950	0.31 [-0.4; 1.0]
		**ZEM 25**	12[O]	128.60 ± 89.54	>0.9999	0.07 [-0.6; 0.8]
		**MES**	12	117.41 ± 31.50	>0.9999	-0.11 [-0.8; 0.6]
**DOPAC/DA**	**$X^2(4) = 23.01, p = 0.0001\text{***}$**	**UCMS**	24[R]	0.73 ± 0.41		
		**ESC**	11	1.04 ± 0.25	**0.0332**	**0.82 [0.1; 1.6]**
		**ZEM 12.5**	12	0.77 ± 0.18	>0.9999	0.12 [-0.6; 0.8]
		**ZEM 25**	12[O]	0.55 ± 0.2	0.6958	-0.5 [-1.2; 0.2]
		**MES**	12[R]	1.13 ± 0.24	**0.0051****	**1.07 [0.4; 1.8]**
**NA (ng/g wet brain)**	$X^2(4) = 3.15, p = 0.5334$	**UCMS**	24[R]	4637 ± 2215		
		**ESC**	11	5259 ± 2598	>0.9999	0.26 [-0.5; 1.0]
		**ZEM 12.5**	12	3509 ± 1510	>0.9999	-0.55 [-1.3; 0.2]
		**ZEM 25**	12	4132 ± 1681	>0.9999	-0.24 [0.9; 0.5]
		**MES**	12[O]	4897 ± 2675	>0.9999	0.11 [-0.6; 0.8]
**Tyrosine (TYR) (ng/g wet brain)**	$X^2(4) = 3.45, p = 0.4859$	**UCMS**	24[O]	106.10 ± 38.51		
		**ESC**	11	121.71 ± 36.95	0.5402	0.4 [-0.3; 1.1]
		**ZEM 12.5**	12[O]	97.19 ± 36.10	>0.9999	-0.23 [-0.9; 0.5]
		**ZEM 25**	12	98.56 ± 23.06	>0.9999	-0.22 [-0.9; 0.5]
		**MES**	12[O]	112.80 ± 38.75	>0.9999	0.17 [-0.5; 0.9]
**3-CLT (ng/g wet brain)**	$X^2(4) = 4.03, p = 0.4022$	**UCMS**	24[O]	93.17 ± 32.13		
		**ESC**	11	106.4 ± 33.70	0.7122	0.4 [-0.3; 1.1]
		**ZEM 12.5**	12[O]	82.78 ± 30.75	>0.9999	-0.32 [-1.0; 0.4]
		**ZEM 25**	12	90.31 ± 25.98	>0.9999	-0.09 [-0.8; 0.6]
		**MES**	12[O]	105.8 ± 35.11	0.8198	0.37 [-0.3; 1.1]
**3-CLT/TYR**	**$X^2(4) = 15.73, p = 0.0034\text{**}$**	**UCMS**	24[R]	0.88 ± 0.06		
		**ESC**	11	0.87 ± 0.05	>0.9999	-0.2 [-1.0; 0.5]
		**ZEM 12.5**	12	0.86 ± 0.02	0.8529	-0.57 [-1.3; 0.1]
		**ZEM 25**	12	0.91 ± 0.08	>0.9999	0.34 [-0.4; 1.1]
		**MES**	12	0.94 ± 0.05	**0.0099**	**1.07 [0.4; 1.8]**
**GSH (ng/g wet brain)**	$X^2(4) = 0.49, p = 0.9740$	**UCMS**	24	80.96 ± 25.05		
		**ESC**	11	85.84 ± 16.81	>0.9999	0.21 [-0.5; 0.9]
		**ZEM 12.5**	12[O]	76.18 ± 26.04	>0.9999	-0.18 [-0.9; 0.5]
		**ZEM 25**	12	80.32 ± 29.86	>0.9999	-0.02 [-0.7; 0.7]
		**MES**	12	83.41 ± 12.98	>0.9999	0.11 [-0.6; 0.8]
**GSSG (ng/g wet brain)**	**$X^2(4) = 10.78, p = 0.0292\text{*}$**	**UCMS**	24[O]	142.70 ± 71.47		
		**ESC**	11[O]	124.01 ± 24.00	>0.9999	-0.3 [-1.0; 0.4]
		**ZEM 12.5**	12[O]	131.10 ± 42.16	>0.9999	-0.18 [-0.9; 05]

		**ZEM 25**	12	157.51 ± 34.56	0.2606	0.23 [-0.5; 0.9]
		**MES**	12	107.10 ± 45.47	0.3031	-0.54 [-1.3; 0.2]
**GSSG/GSH**	**$X^2(4) = 12.26$, $p = 0.0155$***	**UCMS**	24	1.90 ± 0.99		
		**ESC**	11	1.46 ± 0.24	>0.9999	-0.52 [-1.3; 0.2]
		**ZEM 12.5**	12	1.75 ± 0.37	>0.9999	-0.17 [-0.9; 0.5]
		**ZEM 25**	12	2.12 ±0.58	0.1928	0.25 [-0.4; 0.9]
		**MES**	12	1.30 ± 0.54	0.3304	-0.68 [-1.4; 0.0]
**HIPPOCAMPUS**						
**PDE4B (ng/ml)**	**$X^2(4) = 27.47$, $p = <0.0001$********	**UCMS**	20	1.2± 0.17		
		**ESC**	10	1.07 ± 0.13	0.4995	-0.83 [-1.6; -0.1]
		**ZEM 12.5**	10	0.90 ± 0.10	**<0.0001**	**-2.02 [-3.0; -1.1]**
		**ZEM 25**	10	1.09 ± 0.11	0.7179	-0.73 [-1.5; 0.0]
		**MES**	10	0.97 ± 0.06	**0.0013**	**-1.61 [-2.5; -0.8]**
**5-HT (ng/g wet brain)**	$X^2(4) = 6.91$, $p = 0.1409$	**UCMS**	24 ^{O}	90.25 ± 81.45		
		**ESC**	11	80.15 ± 25.87	>0.9999	-0.14 [-0.9; 0.6]
		**ZEM 12.5**	12 ^{O}	115.10 ± 116.40	0.4816	0.26 [-0.4; 1.0]
		**ZEM 25**	12 ^{O}	129.50 ± 159.40	>0.9999	0.34 [-0.4; 1.0]
		**MES**	12 ^{O}	122.70 ± 92.72	0.0723	0.37 [-0.3; 1.1]
**5-HIAA (ng/g wet brain)**	**$X^2(4) = 10.24$, $p = 0.0365$***	**UCMS**	24	279.51 ± 60.61		
		**ESC**	11	315.90 ± 102.20	>0.9999	0.47 [-0.3; 1.2]
		**ZEM 12.5**	12	358.71 ± 82.17	**0.0291**	**1.13 [0.4; 1.9]**
		**ZEM 25**	12	292.12 ± 93.72	>0.9999	0.17 [-0.5; 0.9]
		**MES**	12	264.70 ± 50.87	>0.9999	-0.25 [-1.0; 0.4]
**5-HIAA/5-HT**	$X^2(4) = 6.39$, $p = 0.1718$	**UCMS**	24	3.97 ± 1.49		
		**ESC**	11	4.33 ± 1.92	>0.9999	0.22 [-0.5; 0.9]
		**ZEM 12.5**	12	4.15 ± 1.63	>0.9999	0.12 [-0.6; 0.8]
		**ZEM 25**	12	4.06 ± 2.74	>0.9999	0.04 [-0.7; 0.7]
		**MES**	12	2.77 ± 1.20	0.1167	-0.83 [-1.6; -0.1]
**DA (ng/g wet brain)**	$X^2(4) = 5.63$, $p = 0.2288$	**UCMS**	24 ^{O}	67.02 ± 26.81		
		**ESC**	11	60.03 ± 13.30	>0.9999	-0.29 [-1.0; 0.4]
		**ZEM 12.5**	12 ^{O}	70.09 ± 18.57	0.8783	0.12 [-0.8; 0.8]
		**ZEM 25**	12	53.87 ± 17.51	0.7016	-0.53 [-1.3; 0.2]
		**MES**	12 ^{O}	68.16 ± 42.70	>0.9999	0.03 [-0.7; 0.7]
**DOPAC (ng/g wet brain)**	$X^2(4) = 7.44$, $p = 0.1145$	**UCMS**	24 ^{O}	85.95 ± 31.55		
		**ESC**	11	76.40 ± 16.03	>0.9999	-0.34 [-1.1; 0.4]
		**ZEM 12.5**	12 ^{O}	90.86 ± 28.76	>0.9999	0.16 [-0.5; 0.9]
		**ZEM 25**	12	73.86 ± 31.67	0.7783	-0.37 [-1.1; 0.3]
		**MES**	12 ^{O}	67.93 ± 19.58	0.1906	-0.62 [-1.3; 0.1]
**DOPAC/DA**	$X^2(4) = 3.79$, $p = 0.4355$	**UCMS**	24 ^{O}	1.33 ± 0.38		
		**ESC**	11	1.26 ± 0.20	>0.9999	-0.21 [-0.9; 0.5]
		**ZEM 12.5**	12	1.37 ± 0.54	>0.9999	0.09 [-0.6; 0.8]

		**ZEM 25**	12	1.38 ± 0.36	>0.9999	0.12 [-0.6; 0.8]
		**MES**	12	1.12 ± 0.31	0.4871	-0.58 [-1.3; 0.1]
**NA (ng/g wet brain)**	**$X^2(4) = 16.39$, *p* = 0.0025****	**UCMS**	24[O]	3634 ± 1895		
		**ESC**	11	2753 ± 1043	0.7957	-0.51 [-1.3; 0.2]
		**ZEM 12.5**	12	3579 ± 1083	>0.9999	-0.03 [-0.7; 0.7]
		**ZEM 25**	12[O]	6650 ± 7545	>0.9999	0.65 [-0.1; 1.4]
		**MES**	12	1948 ± 690.1	**0.0074**	**-1.01 [-1.8; -0.3]**
**GSH (ng/g wet brain)**	$X^2(4) = 6.72$, *p* = 0.1516	**UCMS**	24	81.91 ± 20.76		
		**ESC**	11	82.26 ± 15.68	>0.9999	0.02 [-0.7; 0.7]
		**ZEM 12.5**	12	72.41 ± 6.40	0.2202	-0.53 [-1.2; 0.2]
		**ZEM 25**	12	75.64 ± 22.80	>0.9999	-0.29 [-1.0; 0.4]
		**MES**	12	86.80 ± 7.81	>0.9999	0.27 [-0.4; 0.9]
**GSSG (ng/g wet brain)**	**$X^2(4) = 21.03$, *p* = 0.0003*****	**UCMS**	23[R]	187.5 ± 71.39		
		**ESC**	11	129.8 ± 26.06	**0.0200**	**-0.92 [-1.7; -0.2]**
		**ZEM 12.5**	12[O]	138.4 ± 36.82	**0.0379**	**-0.77 [-1.5; -0.1]**
		**ZEM 25**	12[O]	191.7 ± 49.23	0.6373	0.06 [-0.6; 0.8]
		**MES**	12	146.3 ± 16.30	0.4649	-0.68 [-1.4; 0.0]
**GSSG/GSH**	**$X^2(4) = 16.89$, *p* = 0.002****	**UCMS**	24	2.58 ± 1.13		
		**ESC**	11[O]	1.59 ± 0.25	**0.0129**	**-1.0 [-1.8; -0.3]**
		**ZEM 12.5**	12[O]	1.90 ± 0.37	>0.9999	-0.7 [-1.4; 0.0]
		**ZEM 25**	12	2.74 ± 1.00	0.6542	0.14 [-0.5; 0.8]
		**MES**	12	1.69 ± 0.21	0.3818	-0.92 [-1.7; -0.2]
PLASMA						
**TNF-α (pg/ml)**	**$X^2(4) = 14.76$, *p* = 0.0052****	**UCMS**	20	7.80 ± 3.30		
		**ESC**	10	3.89 ± 3.12	0.0678	-1.17 [-2.0; -0.4]
		**ZEM 12.5**	10	8.89 ± 1.32	>0.9999	0.38 [-0.4; 1.1]
		**ZEM 25**	10	10.45 ± 1.94	0.2081	0.88 [0.1; 1.7]
		**MES**	10	8.70 ± 5.09	>0.9999	0.22 [-0.5; 1.0]
**IL-10 (pg/ml)**	$X^2(4) = 5.66$, *p* = 0.2263	**UCMS**	20	17.81 ± 8.68		
		**ESC**	10	19.83 ± 10.75	>0.9999	0.21 [-0.6; 1.0]
		**ZEM 12.5**	10	36.46 ± 20.52	0.1154	1.33 [0.5; 2.2]
		**ZEM 25**	10	23.40 ± 13.33	>0.9999	0.52 [-0.2; 1.3]
		**MES**	10	18.23 ± 8.72	>0.9999	0.05 [-0.7; 0.8]

Table 5. Summary two-way ANOVA results investigating sex differences in behaviour following UCMS exposure. $p < 0.05$. PND – Postnatal day; UCMS – Saline-treated stress group; ESC – Escitalopram; ZEM 12.5 and 25 - Zembrin®; MES – Mesembrine.

**Parameter**	**2-Way ANOVA: *p*-values**			**Group differences**	
	**Interaction**	**Sex**	**Stress**		
**SUCROSE PREFERENCE TEST (SPT)**					
**Sucrose preference PND 50**	0.2098	0.8261	0.2439	ns	
**Sucrose preference PND 64**	**0.0086****	0.1774	0.3214	Males:UCMS vs. Females:UCMS	*p* = 0.0335*
**Sucrose preference PND 78**	**0.0078****	0.6005	0.1923	Males:Ctrl vs. Males:UCMS	*p* = 0.0341*
**Sucrose preference PND 92**	**0.0089****	0.3222	0.4616	ns	
**Sucrose preference PND 101**	**0.0248***	**0.0065****	**0.0309***	Males:Ctrl vs. Males:UCMS<br>Males:UCMS vs. Females:Ctrl<br>Males:UCMS vs. Females:UCMS	*p* = 0.0145*<br>*p* = 0049**<br>*p* = 0.004**
**OPEN FIELD TEST (OFT)**					
**Distance (cm)**	0.1715	0.7784	**0.0004*****	Males:Ctrl vs. Males:UCMS<br>Males:UCMS vs. Females:Ctrl	*p* = 0.0039**<br>*p* = 0.0356*
**Center duration (s)**	0.6360	0.7740	0.2396	ns	
**Corner duration (s)**	0.2538	**0.0014****	0.6275	Males:UCMS vs. Females:UCMS	0.0048*
**FORCED SWIM TEST (FST)**					
**Swimming (s)**	0.2643	0.5777	**0.0004*****	Males:Ctrl vs. Males:UCMS	0.0022**
**Immobility (s)**	0.6810	0.4809	**0.0002*****	Males:Ctrl vs. Males:UCMS<br>Females: Ctrl vs Females: UCMS	0.0056**<br>0.0264*
**Struggling (s)**	**0.0049****	0.5060	0.3135	Females:Ctrl vs. Females:UCMS	0.0432*
**BARNES MAZE (BM)**					
**Distance (cm)**	0.6816	0.3406	0.7384	ns	
**Primary error rate**	**0.0192***	0.6148	0.0729	Males:Ctrl vs. Males:UCMS	
**Edge exploration (head dips)**	**0.0072****	0.5865	0.1246	Females:Ctrl vs. Females:UCMS	
**Outer/inner duration**	0.5619	0.0511	**0.0400***	ns	

Table S6. Summary of two-way ANOVA results investigating sex differences in neurochemical markers following UCMS exposure. $p < 0.05$. 3-CLT – 3-chlorotyrosine; 3-CLT/TYR – 3-CLT turnover rate; 5-HIAA – 5-Hydroxyindoleacetic acid; 5-HT – serotonin; 5-HIAA/5-HT – serotonin turnover rate; DA –- dopamine; DOPAC – 3.4-dihydroxyphenylacetic acid; DOPAC/DA – dopamine turnover rate; ESC – Escitalopram; MES – Mesembrine; NA – noradrenaline; PDE4B – phosphodiesterase 4B; PND – postnatal day; SD – standard deviation; UCMS – saline-treated stress group; TYR – tyrosine; ZEM 12.5 and 25 - Zembrin® 12.5 and 25 mg/kg.

**Parameter**	**2-Way ANOVA: *p*-values**			**Group differences**	
	**Interaction**	**Sex**	**Stress**		
**FRONTAL CORTEX**					
**PDE4B (ng/ml)**	0.1318	0.6517	0.3462	ns	
**5-HT (ng/g wet brain)**	**0.0192***	0.6518	0.3531	ns	
**5-HIAA (ng/g wet brain)**	0.2385	**0.0295***	**0.0081****	Ctrl Females vs. UCMS: Females	*p* = 0.0149*
**5-HIAA/5-HT**	**0.0262***	0.2770	0.1262	ns	
**DA (ng/g wet brain)**	0.0544	0.9473	0.4068	ns	
**DOPAC (ng/g wet brain)**	**0.0016****	0.7179	0.4763	Females:Ctrl vs. Females:UCMS	*p* = 0.0332
**DOPAC/DA**	0.9049	0.7754	0.8362	ns	
**NA (ng/g wet brain)**	0.8744	0.7942	0.1645	ns	
**TYR (ng/g wet brain)**	0.7775	0.7862	0.8211	ns	
**3-CLT (ng/g wet brain)**	0.8813	0.5251	0.3300	ns	
**3-CLT/TYR**	0.9813	0.1569	**0.0057****	ns	
**GSH (ng/g wet brain)**	0.5504	0.4647	0.2847	ns	

**GSSG (ng/g wet brain)**	0.7076	0.3074	**0.5341**	ns	
**GSSG/GSH**	0.5054	**0.0361**	0.6074	ns	
**HIPPOCAMPUS**					
**PDE4B (ng/ml)**	0.0938	0.3224	0.0536	ns	
**5-HT (ng/g wet brain)**	0.0925	0.1574	0.8740	ns	
**5-HIAA (ng/g wet brain)**	0.9245	0.4669	0.2596	ns	
**5-HIAA/ 5-HT**	0.1748	0.6837	0.3550	ns	
**DA (ng/g wet brain)**	0.9966	0.9126	0.4090	ns	
**DOPAC (ng/g wet brain)**	0.8254	0.6835	0.2852	ns	
**DOPAC/DA**	0.1930	0.4080	0.9985	ns	
**NA (ng/g wet brain)**	0.0651	0.0593	0.0715	ns	
**TYR (ng/g wet brain)**	0.7383	0.2084	0.5265	ns	
**3-CLT (ng/g wet brain)**	0.9255	0.1623	0.2933	ns	
**3-CLT/TYR**	0.4689	0.4115	0.2526	ns	
**GSH (ng/g wet brain)**	0.0917	**0.0436**	0.8129	Males UCMS vs. Females UCMS	0.0202
**GSSG**	0.2136	**0.0304**	0.5894	Males UCMS vs. Females UCMS	0.0346

**(ng/g wet brain)**					
**GSSG/GSH**	0.9390	0.5376	0.5809	ns	
**PLASMA**					
**IL-10 (pg/ml)**	0.1039	**0.0378**	0.1295	Male Ctrl. Vs. Female Ctrl.	0.0207
**TNF-α (pg/ml)**	0.5670	0.9869	0.7314	ns	

Table S7. Summary of two-way ANOVA results investigating sex differences in behaviour following treatment of UCMS-exposed Wistar rats. $p < 0.05$. PND – Postnatal day; SD – standard deviation; UCMS – Saline-treated stress group; ESC – Escitalopram; ZEM 12.5 and 25 - Zembrin®; MES – Mesembrine.

Parameter	Two-way ANOVA			Bonferroni multiple comparison	
	Interaction	Sex	Treatment	Groups	*p*-value
**SUCROSE PREFERENCE TEST (SPT)**					
**Sucrose preference PND50**	0.3042	0.8810	0.2825	ns	
**Sucrose preference PND64**	0.0506	0.1829	0.8333	ns	
**Sucrose preference PND78**	**0.0126**	0.1067	0.1430	UCMS:Males vs. ESC:Females<br>UCMS:Males vs. MES:Males	0.0484<br>0.0124
**Sucrose preference PND92**	0.2057	0.3393	**0.0442**	UCMS Males vs. UCMS Females	0.0134
**Sucrose preference PND101**	**0.0397**	**0.0294**	0.0862	UCMS:Males vs. UCMS:Females<br>UCMS:Males vs. ZEM12.5:Males<br>UCMS:Males vs. ZEM12.5:Females<br>UCMS:Males vs. ZEM25:Females	0.0053<br>0.0128<br>0.0157<br>0.0233
**OPEN FIELD TEST (OFT)**					
**Distance (cm)**	0.1907	**0.0480**	0.6183	ns	
**Center duration (s)**	0.5781	**0.0071**	0.1796	Males: ESC vs. ZEM25	0.0458
**Corner duration (s)**	0.5483	**0.0270**	**0.0025**	Males UCMS vs. Females UCMS	0.0079
**FORCED SWIM TEST (FST)**					
**Swimming (s)**	0.4414	**0.0004**	0.5608	Males: ZEM12.5 vs. ZEM25	0.0078
**Immobility (s)**	0.8348	**0.0002**	0.6690	Males: ZEM12.5 vs. ZEM25<br>Females: ZEM12.5 vs. MES	0.0154<br>0.0284
**Struggling (s)**	0.0744	0.1278	0.6272	ns	
**BARNES MAZE (BM)**					
**Distance (cm)**	0.0952	**0.0019**	**0.0335**	Females: UCMS vs. MES<br>Females: ESC vs. MES	0.0214<br>0.0062

				Females: ZEM12.5 vs. MES<br>Females: ZEM25 vs. MES<br>Males MES vs. Females UCMS	0.0022<br>0.0467<br>0.0094
**Primary error rate**	0.1498	0.7086	0.7034	ns	
**Edge exploration (head dips)**	0.0972	**0.0119**	0.2392	Females: UCMS vs. MES<br>Females: ESC vs. MES	0.0232<br>0.0169
**Outer/inner duration**	0.2985	0.5079	**0.0087**	Males: ESC vs. Females ESC	0.0254

Table S8. Summary of two-way ANOVA results investigating sex differences in neurochemistry following treatment of UCMS-exposed Wistar rats. $p < 0.05$. 3-CLT – 3-chlorotyrosine; 3-CLT/TYR – 3-CLT turnover rate; 5-HIAA – 5-Hydroxyindoleacetic acid; 5-HT – serotonin; 5-HIAA/5-HT – serotonin turnover rate ; 95% CI – 95% confidence intervals; DA –- dopamine; DOPAC – 3.4-dihydroxyphenylacetic acid; DOPAC/DA – dopamine turnover rate; ESC – Escitalopram; MES – Mesembrine; NA – noradrenaline; PDE4B – phosphodiesterase 4B; PND – postnatal day; SD – standard deviation; UCMS – saline-treated stress group; TYR – tyrosine; ZEM 12.5 and 25 - Zembrin® 12.5 and 25 mg/kg.

**Parameter**	**Two-way ANOVA**			**Bonferroni multiple comparison**	
	**Interaction**	**Sex**	**Treatment**	**Groups**	***p*-value**
**FRONTAL CORTEX (FC)**					
**PDE4B (ng/ml)**	0.1063	**<0.0001**	0.9555	Males: UCMS vs. ZEM12.5<br>Males: ESC vs. ZEM12.5<br>Males: ZEM12.5 vs. MES<br>Females: UCMS vs. ZEM12.5<br>Females: ESC vs. ZEM12.5<br>Females: ESC vs. ZEM25	<0.0001<br>0.0044<br>0.0013<br>0.0171<br><0.0001<br>0.0402
**5-HT (ng/g wet brain)**	0.8824	0.7270	0.6762	ns	
**5-HIAA (ng/g wet brain)**	**0.0168**	**<0.0001**	0.1458	UCMS:Males vs. ESC:Males<br>UCMS:Females vs. ESC:Males<br>ESC:Males vs. ESC:Females<br>ESC:Males vs. ZEM25:Males<br>ESC:Males vs. ZEM25:Females<br>ESC:Males vs. MES:Males<br>ESC:Males vs. MES:Females	0.0017<br>0.0001<br>0.0250<br>0.0004<br>0.0076<br>0.0002<br>0.0007
**5-HIAA/5-HT**	0.5493	**0.0087**	**0.0120**	Males UCMS vs. Females UCMS	0.0193
**DA (ng/g wet brain)**	0.9602	0.1062	0.1299	ns	
**DOPAC (ng/g wet brain)**	0.3722	0.6809	0.0627	ns	
**DOPAC/DA**	0.1880	**<0.0001**	0.3239	Females: UCMS vs. ESC<br>Females: UCMS vs. MES<br>Females: ESC vs. ZEM25<br>Females: ZEM25 vs. MES	0.0225<br>0.0075<br>0.0005<br>0.0002
**NA**	**0.0477**	0.3044	**0.0020**	Males ESC vs. Females ESC	0.0467

**(ng/g wet brain)**					
**TYR (ng/g wet brain)**	0.1995	0.4788	**0.0359**	ns	
**3-CLT (ng/g wet brain)**	0.0711	0.2763	**0.0068**	ns	
**3-CLT/TYR**	**0.0036**	**0.0002**	**0.0008**	UCMS: Males vs. ZEM25:Females<br>ESC: Males vs. ZEM25:Females<br>ZEM12.5: Males vs. ZEM25: Females<br>ZEM12.5: Males vs. MES: Females<br>ZEM12.5: Females vs. ZEM25: Females<br>ZEM25: Males vs. ZEM25: Females<br>ZEM25: Males vs. MES: Males<br>ZEM25: Males vs. MES: Females	0.0013<br>0.0031<br>0.0011<br>0.0500<br>0.0019<br>0.0001<br>0.0118<br>0.0085
**GSH (ng/g wet brain)**	**0.0002**	0.8479	0.1075	ZEM12.5: Females vs. ZEM25: Females<br>ZEM25: Males vs. ZEM25: Females	0.0357<br>0.0006
**GSSG (ng/g wet brain)**	0.5361	0.1435	0.0553	ns	
**GSSG/GSH**	0.1209	**0.0233**	0.8576	Males ZEM25 vs. Male MES	0.00666
**HIPPOCAMPUS (HC)**					
**PDE4B (ng/ml)**	0.8192	**<0.0001**	0.5771	Males UCMS vs. Males ZEM12.5<br>Males UCMS vs. Males MES<br>Female UCMS vs. ZEM12.5<br>Female UCMS vs. MES	<0.0001<br>0.0179<br>0.0093<br>0.0288
**5-HT (ng/g wet brain)**	0.2713	0.6175	**0.0074**	Males ZEM25 vs Females ZEM25	0.0281
**5-HIAA (ng/g wet brain)**	**0.0034**	**0.0093**	0.6775	ESC:Males vs. MES:Males<br>ZEM12.5:Females vs. MES:Males	0.0101<br>0.0261
**5-HIAA/5-HT**	**0.0043**	**0.0794**	**<0.0001**	UCMS:Males vs. MES:Males<br>UCMS:Females vs. ZEM25:Males<br>ESC:Males vs. ZEM25:Females<br>ESC:Males vs. MES:Males<br>ZEM12.5:Females vs. MES:Females<br>ZEM25:Males vs. ZEM25:Females	0.0288<br>0.0280<br>0.0310<br>0.0168<br>0.0054<br>0.0027
**DA (ng/g wet brain)**	0.3453	0.5087	0.2270	ns	
**DOPAC (ng/g wet brain)**	0.1880	0.1779	0.0839	ns	
**DOPAC/DA**	0.1019	0.3779	0.6152	ns	
**NA (ng/g wet brain)**	0.0663	**0.0114**	0.1471	Females UCMS vs. Females ZEM25<br>Females ESC vs. Females ZEM25<br>Females: ZEM12.5 vs. ZEM25<br>Females: ZEM25 vs. MES	0.0043<br>0.0085<br>0.0113<br>0.0006

**TYR (ng/g wet brain)**	0.6503	0.0549	0.6569	ns	
**3-CLT (ng/g wet brain)**	0.5240	0.9358	0.8185	ns	
**3-CLT/TYR**	0.7182	**0.0043**	0.0622	Females: UCMS vs. ZEM25<br>Females: ESC vs. ZEM25<br>Females: ZEM12.5 vs. ZEM25	0.0056<br>0.0083<br>0.0238
**GSH (ng/g wet brain)**	0.1209	**0.0233**	0.8576	Males: ZEM25 vs. MES	0.0066
**GSSG (ng/g wet brain)**	0.0857	**0.0013**	0.7699	Females: UCMS vs. ESC<br>Females: UCMS vs. ZEM12.5	0.0155<br>0.0035
**GSSG/GSH**	**0.0133**	**0.0001**	0.0667	ESC:Males vs. ZEM25:Males<br>ESC:Females vs. ZEM25:Males<br>ZEM12.5:Males vs. ZEM25:Males<br>ZEM12.5:Females vs. ZEM25:Males<br>ZEM25:Males vs. ZEM25:Females<br>ZEM25:Males vs. MES:Males<br>ZEM25:Males vs. MES:Females	0.0065<br>0.0005<br>0.0394<br>0.0040<br>0.0159<br>0.0010<br>0.0071
**PLASMA**					
**IL-10 (pg/ml)**	**0.0034**	**<0.0001**	**<0.0001**	UCMS:Males vs. ZEM12.5:Males<br>UCMS:Females vs. ZEM12.5:Males<br>UCMS:Females vs. ZEM25:Males<br>ESC:Males vs. ZEM12.5:Males<br>ESC:Females vs. ZEM12.5:Males<br>ESC:Females vs. ZEM25:Males<br>ZEM12.5:Males vs. ZEM12.5:Females<br>ZEM12.5:Males vs. ZEM25:Females<br>ZEM12.5:Males vs. MES:Males<br>ZEM12.5:Males vs. MES:Females<br>ZEM25:Males vs. ZEM25:Females<br>ZEM25:Males vs. MES:Females	<0.0001<br><0.0001<br>0.0214<br>0.0028<br><0.0001<br>0.0417<br>0.0012<br><0.0001<br>0.0074<br><0.0001<br>0.0039<br>0.0029
**TNF-α (pg/ml)**	0.7682	**0.0010**	0.5653	Males: ESC vs. ZEM25	0.0069
