# Supplementary material for: Neuro-Inflammatory and Behavioral Changes Are Selectively Reversed by Sceletium tortuosum (Zembrin®) and Mesembrine in Male Rats Subjected to Unpredictable Chronic Mild Stress
Source: Cells. 2025 Jul 4;14(13):1029. doi: 10.3390/cells14131029 (PMC12249403; doi:10.3390/cells14131029)
Supplement: Supplementary file 1 [file cells-14-01029-s001.zip › Supplementary material B - HPLC-EC method for oxidative stress markers.pdf]

SUPPLEMENTARY MATERIAL B: METHOD FOR HPLC ELECTROCHEMICAL DETECTION OF OXIDATIVE STRESS MARKERS IN RAT BRAIN TISSUE SAMPLES

Sceletium tortuosum (Zembrin®) and mesembrine reverse increased phosphodiesterase 4B as well as associated anhedonia and anxiety-related hyperactivity in male rats subjected to unpredictable chronic mild stress

*Johané Gericke[a], Stephan Steyn[a] Francois Viljoen[a], Brian H. Harvey[a,b,c]**

[a]*Centre of Excellence for Pharmaceutical Sciences, Faculty of Health Sciences, North-West University, Potchefstroom, RSA*

[b]*South African Medical Research Council Unit on Risk and Resilience in Mental Disorders, Department of Psychiatry and Neuroscience Institute, University of Cape Town, Cape Town, RSA*

[c]*The Institute for Mental and Physical Health and Clinical Translation, School of Medicine, Deakin University, Geelong 3220, AUS*

*Corresponding author: Brian.harvey@nwu.ac.za; Tel: +27 18 299 2234

ORCiD of Authors

Johané Gericke: https://orcid.org/0000-0003-0038-3374

Stephan Steyn: https://orcid.org/0000-0002-0023-9711

Francois Viljoen: https://orcid.org/0000-0002-0145-7104

Brian H Harvey: https://orcid.org/0000-0002-9864-7894

Oxidative stress marker analyses

A modified high performance liquid chromatography with electochemical detection (HPLC-EC) method of Bayram et.al. [1] was utilized for the detection and quantification of reduced (GSH) and oxidized (GSSG) glutathione, 3-chlorotyrosine (3-CLT) and tyrosine (TYR) in male and female Wistar rat brain samples, collected on postanatal day 101. Other references [1-9] were consulted as well to develop the method.

1. **Sample preparation**

The samples collected for this study was prepared for the quantification of the monoamines and their metabolites according to the method by Viljoen et al. [10], which was also compatible with the method described in this document. The samples were thus prepared according to this method, frozen, and later used for the oxidative stress analyses described in this document. The following is an exerpt of the sample preparation method from Viljoen et al. [10].

Solution A

Solution A (consisting of 0.1 M perchloric acid; 0.5 mM sodium metabisulphite and 0.3 mM Ethylenediaminetetraacetic acid disodium salt) was used as the preparation solvent for all samples. The purpose of this solution was to keep the markers protected from auto-oxidation and for the precipitation of proteins in the biological samples, thus keeping the samples stable for longer.

Sample preparation of brain tissue samples [11]

Brain tissue samples of different brain areas (hippocampus and frontal cortex), collected from laboratory animals via dissection, were transferred to 1.5 ml Eppendorf tubes, immediately snap frozen with liquid nitrogen and stored at -80 .C until the day of analysis. On the day of analysis, the brain tissue sample was weighed and 1 ml of solution A was added to the tube. The tissue in the tube was then ruptured by sonication (2 x 12 s, at an amplitude of 14 µ. The tube was placed in ice for 20 min to complete perchlorate precipitation of proteins and extraction of analytes from the brain tissue. The sample was then centrifuged at 4˚C for 25 min at 20 817 rcf. The supernatant fluid (tissue extract) was removed and pipetted into 2 ml amber Eppendorf tube. The pH of the sample was adjusted to 5.0 with the addition of 1 drop of 10 M potassium acetate. An aliquot of 200 µl of the tissue extract, or standard, was pipetted into 1.5 ml Eppendorf tube and 20 µl of the internal standard, 5-HMT, was added to the sample. The rest of the extracted brain tissue sample was stored at -80 °C. The final sample was vortexed and centrifuged for 5 min at 20 817 rcf and transferred to HPLC vial insert.

2. HPLC electrochemical detection method

2.1. Chemicals, Standards, mobile phase, and instrument setup

2.1.1. Standards and chemicals

GSH, GSSG, Tyrosine and 3-Chlorotyrosine obtained from Sigma-Aldrich® (Johannesburg, South Africa). Reagents used for the mobile phase were HPLC grade deionized water, HPLC grade methanol (MeOH), and formic acid (99%), while those used for the preparation of standards and samples were HPLC grade MeOH, acetonitrile (ACN), and formic acid (99%). All of these were obtained from Merck® (Johannesburg, South Africa).

2.1.2. Instrumentation and column

A Venusil ASB C18 column, 4.6 × 250 mm, 5 µm, 300Å (purchased from Bonna-Agela Technologies, USA) analytical HPLC column was used for chromatographic separation. A Dionex Ultimate 3000 UHPLC system was used, which consisted of an autosampler, pump, and column compartment, coupled to a Dionex Ultimate 3000 RS electrochemical detector with a coulometric flow cell (6011 RS ultra–Analytical Cell) and the Chromeleon® Chromatography Management System (obtained from Thermo Fisher Scientific, Waltham, MA USA).

2.1.3. Standard solutions

Approximately 10 mg of GSSG and 1 mg each of GSH, tyrosine and 3-CLT was dissolved in 10 ml of distilled water in amber volumetric flasks. From the stock solutions of each analyte, a series consisting of 6 concentrations were prepared to construct a standard calibration curve and determine the linear range of each analyte.

Table 1. Summary of the method.

**Parameter**	**Method**
**HPLC system**	A Dionex Ultimate 3000 UHPLC system was used, which consisted of an autosampler, pump, and column compartment, coupled to a Dionex Ultimate 3000 RS electrochemical detector with a coulometric flow cell (6011 RS ultra–Analytical Cell) and the Chromeleon® Chromatography

	Management System (obtained from Thermo Fisher Scientific, Waltham, MA USA).	
**Column**	Venusil ASB C18 column, 4.6 × 250 mm, 5 μm, 300Å	
**Mobile phase**	99% 50μM NaH2O4P (5.999 g/l), 1 % Acetonitrile, pH adjusted to ±2.8 with 500μl 85% H_3PO_4	
**HPLC instrumentation settings:**		
Flow rate	0.35 ml/min	
Flow gradient	Time (min)	Flow rate (ml/min)
	0.00	0.350
	16.00	0.350
	16.10	0.750
	35.50	0.750
	35.60	0.350
	40.00	0.350
Injection volume	25 μl	
Run time	40 min	
**Electrochemical detector settings:**		
Cell potential settings	Test electrode 1 (E1): -150 mV (to eliminate background noise)	
	Test electrode 2 (E2): +850 mV (to detect analytes)	
	Detection range: 100 nA	
	Filter: 0.5 seconds	
	Offset: 0%	
	Signal output: 0.1 V	
Data collection rate	20 [Hz]	
Operating mode of coulometric cell	Direct current: μA	

3. Results

3.1. Linearity/calibration curve

The calibration curves constructed were evaluated by means of interpreting respective linear regression values. Linearity was excellent over the respective calibration ranges (Table 2), with the corresponding coefficient of determination (R^2) values consistently greater than 0.95 (Shabir BYG (2005).

Table 2: Linear regression line equation and coefficient of determination (R^2). All calibration curves and linearity were processed in GraphPad Prism (version 10).

Analyte	Concentration range μg/ml	Linear regression equation	R^2	Is slope significantly non-zero?
**GSH**	1.5625; 3.125; 6.25; 12.5; 25.0; 50.0	Y = 30.84*X + 25.14	0.9983	Yes $p \leq 0.0001$
**GSSG**	7.8125; 15.625; 31.25; 62.5; 125.0; 250	Y = 1.748*X + 28.28	0.9938	
**Tyrosine**	1.5625; 3.125; 6.25; 12.5; 25.0; 50.0	Y = 19.53*X - 7.634	0.9987	
**3-Chlorotyrosine**	1.5625; 3.125; 6.25; 12.5; 25.0; 50.0	Y = 16.04*X - 8.828	0.9994	

3.1.1. Quantification and detection limits

The limits of quantification (LOQ) and detection (LOD) were determined by means of mathematical formula, the signal to noise approach, and also on-column with a 25 μl injection volume for all the analytes (Table 3).

Table Error! No text of specified style in document.**:** Limits of quantification and detection results. GSH: Glutathione. GSSG: Glutathione disulphide. LOD: Limit of detection. LOQ: Limit of quantification.

Analyte	Calculation approach		Signal to noise approach	
	LOQ (μg/ml)	***LOD (μg/ml)***	***LOQ (μg/ml)***	***LOD (μg/ml)***
**GSH**	8.15	2.69	1.56	0.78
**GSSG**	28.51	9.41	7.81	3.90
**Tyrosine**	11.46	3.78	1.56	0.78
**3-Chlorotyrosine**	2.79	0.92	1.56	0.78

3.1.2. Precision and accuracy

The precision and accuracy results of the four tested concentrations of each analyte are provided in Table 4. Both precision and accuracy results were within the acceptable criteria ranges set by the method validation parameters. For all four concentrations of the respective analytes, the % RSD (relative standard deviation) for the intra-sample precision was below the required 15% Shabir BYG (2005). The accuracy of all concentration levels for all the analytes tested was between 85% and 113% Shabir BYG (2005).

Table 4: Accuracy and precision results. Where applicable data are presented as mean ± standard deviation. GSH: Glutathione. GSSG: Glutathione disulphide. RSD: Relative standard deviation.

Analyte	Intra-sample (n = 3)		
	Measured Concentration (µg/ml)	*Precision (% RSD)*	*Accuracy (%)*
**GSH**			
1.5625	1.33	3.22	85
6.25	6.48	1.44	103
12.5	13.99	3.23	111
25.0	28.07	1.37	112
**GSSG**			
7.8125	6.79	3.93	87
31.25	30.25	3.96	96
62.5	69.39	2.06	111
125	137.58	0.94	110
**Tyrosine**			
1.5625	1.71	1.38	109
6.25	5.84	4.02	93
12.5	11.24	2.27	89
25.0	23.14	2.59	92
**3-Chlorotyrosine**			
1.5625	1.78	5.77	113
6.25	6.15	3.95	98
12.5	11.72	1.97	93
25.0	23.81	4.68	95

3.2. Chromatographic results

The following figures contains the chromatographic results obtained with the method in two blank samples containing the internal standards for GSH, GSSG, 3-CLT, and TYR (Figures 1 and 2), as well as in a hippocampal (Figure 3) and cortical (Figure 4) rat brain sample. We were able to successfully identify each of the analytes in the samples.

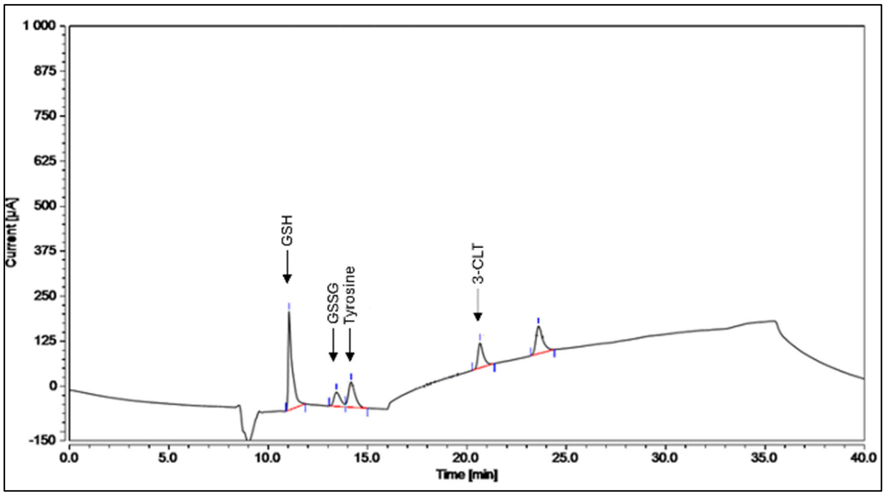

Figure 1. Blank samples with internal standards: GSH, 3-CLT, TYR (1.56 µg/ml), and GSSG (7.81 µg/ml).

Commented [JG1]: Wat is die konsentrasie van die 2 standaarde? STD 1b.

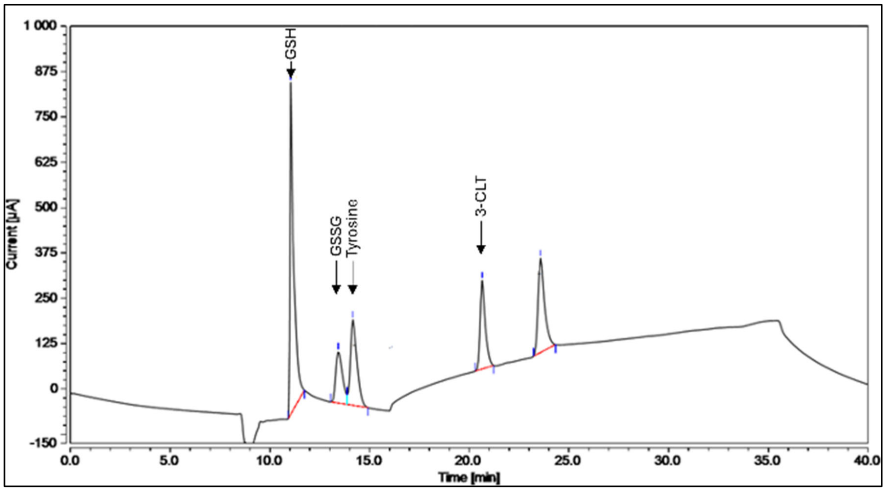

Figure 2. Blank samples with internal standards: GSH, 3-CLT, TYR (12.5 µg/ml), and GSSG (62.5 µg/ml).

Commented [JG2]: Wat is die konsentrasie van die 2 standaarde? STD 1b.

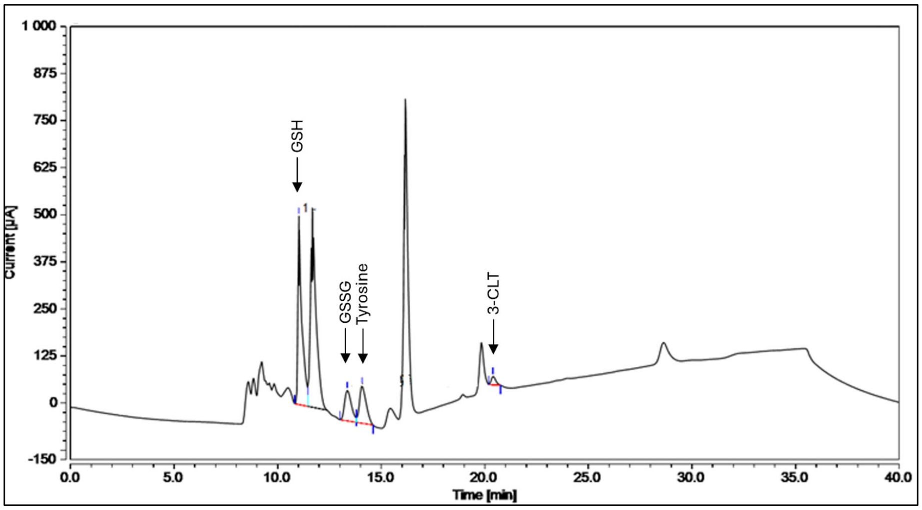

Figure 3. Rat brain tissue – hippocampus (69 mg)

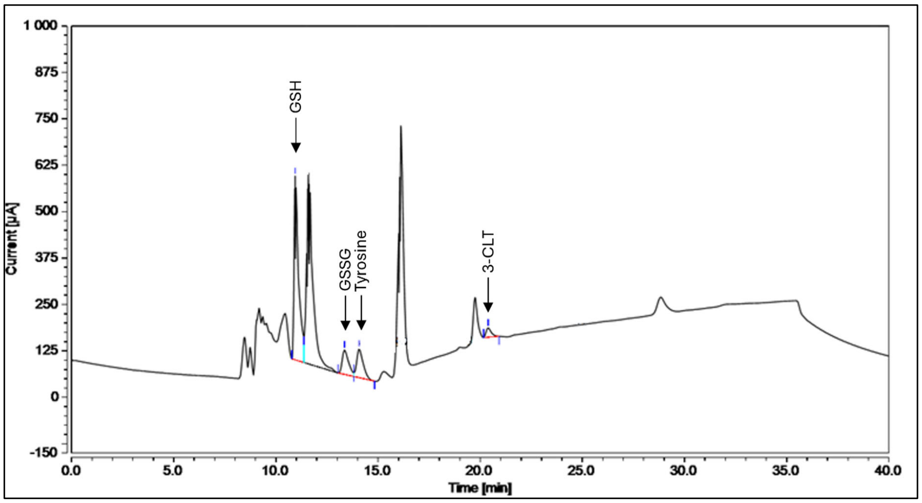

Figure 4. Rat brain tissue – frontal cortex (85.5 mg)

Table 5: Summary of the concentrations (ng/g) of each analyte in the hippocampal and frontal cortex samples analyzed in Figures 3 and 4.

Analyte	Concentration (ng/g)	
	**Hippocampus sample: 69 mg (Fig. 3)**	**Frontal cortex sample: 85.5mg (Fig. 4)**
**GSH**	73.4	45.6
**GSSG**	217.4	96.7
**TYROSINE**	132.2	73.5
**3-CLT**	110.5	63.6

4. References

1. Bayram, B., Rimbach, G., Frank, J., and Esatbeyoglu, T., Rapid method for glutathione quantitation using high-performance liquid chromatography with coulometric electrochemical detection. *Journal of agricultural and food chemistry*, 2014. **62**(2): p. 402-408.
2. Yap, L.-P., Sancheti, H., Ybanez, M.D., Garcia, J., Cadenas, E., and Han, D., Determination of GSH, GSSG, and GSNO using HPLC with electrochemical detection. *Methods in enzymology*, 2010. **473**: p. 137-147.
3. Monostori, P., Wittmann, G., Karg, E., and Túri, S., Determination of glutathione and glutathione disulfide in biological samples: an in-depth review. *Journal of Chromatography*, 2009. **877**(28): p. 3331-3346.
4. Zitka, O., Skalickova, S., Gumulec, J., Masarik, M., Adam, V., Hubalek, J., Trnkova, L., Kruseova, J., Eckschlager, T., and Kizek, R., Redox status expressed as GSH: GSSG ratio as a marker for oxidative stress in paediatric tumour patients. *Oncology letters,* 2012. **4**(6): p. 1247-1253.
5. Lakritz, J., Plopper, C.G., and Buckpitt, A.R., Validated high-performance liquid chromatography-electrochemical method for determination of glutathione and glutathione disulfide in small tissue samples. *Analytical biochemistry*, 1997. **247**(1): p. 63-68.
6. Hensley, K., Williamson, K.S., Maidt, M.L., Prasad Gabbita, S., Grammas, P., and Floyd, R.A., Determination of biological oxidative stress using high performance liquid chromatography with electrochemical detection (HPLC-ECD). *Journal of high resolution chromatography*, 1999. **22**(8): p. 429-437.
7. Crow, J.P. Measurement and significance of free and protein-bound 3-nitrotyrosine, 3-chlorotyrosine, and free 3-nitro-4-hydroxyphenylacetic acid in biologic samples: A high-performance liquid chromatography method using electrochemical detection. *Methods in enzymology*, 1999. **301**: p. 151-160.
8. Potesil, D., Petrlova, J., Adam, V., Vacek, J., Klejdus, B., Zehnalek, J., Trnkova, L., Havel, L., and Kizek, R. Simultaneous femtomole determination of cysteine, reduced and oxidized glutathione, and phytochelatin in maize (Zea mays L.) kernels using high-performance liquid chromatography with electrochemical detection. *Journal of Chromatography*, 2005. **1084**(1-2): p. 134-144.
9. Shabir, G.A., Step-by-step analytical methods validation and protocol in the quality system compliance industry. *Journal of validation technology*, 2005. **10**: p. 314-325.
10. Viljoen, F., Du Preez, J., Wessels, J., and Aucamp, M. HPLC electrochemical detection and quantification of monoamines and their metabolites in rat brain tissue samples*. Die Pharmazie-An International Journal of Pharmaceutical Sciences*, 2018. **73**(10): p. 563-569.
11. Harvey, B.H., Brand, L., Jeeva, Z., and Stein, D.J. Cortical/hippocampal monoamines, HPA-axis changes and aversive behavior following stress and restress in an animal model of post-traumatic stress disorder. *Physiology & behavior,* 2006. **87**(5): p. 881-890.
